# Metallic charge transport in conjugated molecular bilayers

**DOI:** 10.1038/s41928-025-01553-5

**Published:** 2026-01-20

**Authors:** Kuakua Lu, Yun Li, Qijing Wang, Linlu Wu, Xinglong Ren, Xu Chen, Luhao Liu, Yating Li, Xiaoming Xu, Qingkai Zhang, Di Wang, Liqi Zhou, Mingfei Xiao, Sai Jiang, Mengjiao Pei, Haoxin Gong, William Wood, Ian E. Jacobs, Junzhan Wang, Gang Chen, Peng Wang, Zhaosheng Li, Chunfeng Zhang, Xinran Wang, Xu Wu, Yeliang Wang, Wei Ji, Songlin Li, Jingsi Qiao, Yi Shi, Henning Sirringhaus

**Affiliations:** 1 School of Electronic Science and Engineering, National Laboratory of Solid-State Microstructures, Collaborative Innovation Centre of Advanced Microstructures, Nanjing University, Nanjing, China; 2 School of Integrated Circuits, Nanjing University, Suzhou, China; 3 Optoelectronics Group, Cavendish Laboratory, Cambridge, UK; 4 Beijing Key Laboratory of Optoelectronic Functional Materials and Micro-Nano Devices, School of Physics, Renmin University of China, Beijing, China; 5 Key Laboratory of Quantum State Construction and Manipulation (Ministry of Education), Renmin University of China, Beijing, China; 6 Department of Functional Nanosystems, Interdisciplinary Research Center, Liaoning Academy of Materials, Shenyang, China; 7 College of Engineering and Applied Sciences, National Laboratory of Solid-State Microstructures, Collaborative Innovation Center of Advanced Microstructures, Nanjing University, Nanjing, China; 8 School of Physics, Nanjing Institute of Atomic-Level Manufacturing, Nanjing University, Nanjing, China; 9 Institute of Materiobiology, College of Sciences, Shanghai University, Shanghai, China; 10 School of Microelectronics and Control Engineering, Changzhou University, Changzhou, China; 11 School of Physical Science and Technology, ShanghaiTech University, Shanghai, China; 12 Department of Physics, University of Warwick, Coventry, UK; 13 School of Integrated Circuits and Electronics & School of Multidisciplinary Science & School of Physics, Beijing Institute of Technology, Beijing, China

**Keywords:** Electronic devices, Two-dimensional materials

## Abstract

Metallic charge transport of field-induced carriers can be observed in single-crystal silicon over a wide temperature range. Such behaviour is rare in undoped organic semiconductors but is beneficial for engineering devices with advanced performance. Here we report metallic charge transport in conjugated molecular bilayers down to 8 K with an electrical conductivity of up to 245 S cm^−1^ and a Hall mobility larger than 100 cm^2^ V^−1^ s^−1^ at 20 K. We use molecular-crystal bilayers of the organic semiconductor 2-decyl-7-phenyl-[1]benzothieno[3,2-*b*][1]benzothiophene. We infer that this transport behaviour originates from the phenyl bridge coupling between the two molecular layers, which suppresses molecular vibrations and weakens Coulomb interactions. We develop a controlled method for introducing defects, using which we observe a disorder-driven metal–insulator transition in the molecular crystal.

## Abstract (web summary)

Molecular bilayer crystals of an organic semiconductor can exhibit metallic charge transport down to 8 K with an electrical conductivity of up to 245 S cm^−1^, as well as charge carrier mobility values of more than 100 cm^2^ V^−1^ s^−1^ at 20 K.

## Main

In metal–oxide–semiconductor field-effect transistors of covalently bonded semiconductors, such as silicon, it is possible—at sufficiently high gate voltages—to induce a metallic charge transport regime in which the conductivity (*σ*) increases with decreasing temperature (*T*) (∂*σ*/∂*T* < 0) and remains finite as *T* → 0 K (refs. ^1–3^). Such metallic transport is harder to observe in organic semiconductors, which consist of conjugated molecules assembled via weak van der Waals (vdW) forces. These organic materials possess characteristics such as mechanical flexibility, high synthetic tunability and good adaptivity, and can be used to create multifunctional sensing and flexible electronic devices^4,5^. However, compared with silicon, their charge carrier mobilities are particularly lower^6,7^, typically below 20–30 cm^2^ V^−1^ s^−1^. This is because the weak vdW interactions make the charge carriers sensitive to their transport environment. Even in a perfect single crystal with very limited traps, charges experience a dynamically disordered electronic landscape, in which transfer integrals and site energies fluctuate as a result of molecular vibrations; charge transport within such an environment can be understood within the framework of transient localization, which is intermediate between band and hopping transport^8,9^.

Transient localization theory, which considers the electron wavefunction to be pulsating between highly localized and highly delocalized states, predicts that the charge carrier mobility (*μ*) should exhibit a band-like temperature dependence (∂*μ*/∂*T* < 0), that is, increase with decreasing temperature, as the dynamic disorder reduces and the average charge delocalization length becomes longer^8,9^. In practice, however, even in the cleanest molecular crystals, there is usually a very narrow temperature range, typically between around 100–200 K and room temperature, where this predicted mobility increase with decreasing temperature is observed^10–12^. At lower temperatures, most molecular crystals exhibit a transition to a thermally activated transport regime, resulting in a reduction in mobility and *μ* → 0 as *T* → 0. This is attributed to the capturing of mobile charge carriers in shallow trap states, presumed to be due to chemical impurities or structural defects in the bulk of the organic semiconductor or at its interface with the gate dielectric.

The notions of metallic (∂*σ*/∂*T* < 0) and band-like transport (∂*μ*/∂*T* < 0) are closely related to each other, because *σ* = *en*_c_*μ*, where *n*_c_ is the carrier concentration and *e* is the elementary charge. The topic of metallic charge transport in organic semiconductors can be traced back to the observation of electrical conductivity in doped polyacetylene in 1977 (ref. ^13^). In most cases, the observations of such transport behaviour are from doped materials^14–16^, as well as at charge transfer interfaces between molecular crystals^17^, whereas it is indeed rare in undoped organic semiconductors^18^. In studies of organic field-effect transistors (FETs), band-like transport (as an evaluation of the temperature-dependent mobility) has provided an insight into the underpinning charge transport mechanisms^6^. However, metallic charge transport to cryogenic temperatures is technically more difficult to achieve than band-like transport because for band-like transport, only the mobility must increase with decreasing temperature, whereas *n*_c_ may, in fact, decrease.

In FETs, a decrease in carrier concentration commonly manifests itself as an increase in the threshold voltage (*V*_T_) at low temperatures^11,19–22^, as some field-induced carriers are captured in immobile trap states^11,22^. The mobility is often extracted from fits of the transfer characteristics above *V*_T_ and is claimed to be band like even if *V*_T_ is increasing strongly at a low temperature. By contrast, to achieve metallic transport, increases in *V*_T_ and reduction in *n*_c_ must be small. Moreover, metallic transport in molecular-crystal transistors has been observed down to cryogenic temperatures in devices based on the undoped molecular semiconductor 3,11-dioctyldinaphtho[2,3-*d*:2′,3′-*d*′]benzo[1,2-*b*:4,5-*b*′]dithiophene gated by ionic liquids^18^. In such devices, the formation of an ionic double layer on the surface of the crystals allows much higher charge densities to be reached (10^14^ cm^−2^) than in conventional FETs (less than 10^13^ cm^−2^), which makes it easier to fill the trap states. Whether such metallic charge transport is also achievable at lower carrier concentrations in a molecular FET with a conventional gate dielectric remains an open question.

**Fig. 1:**
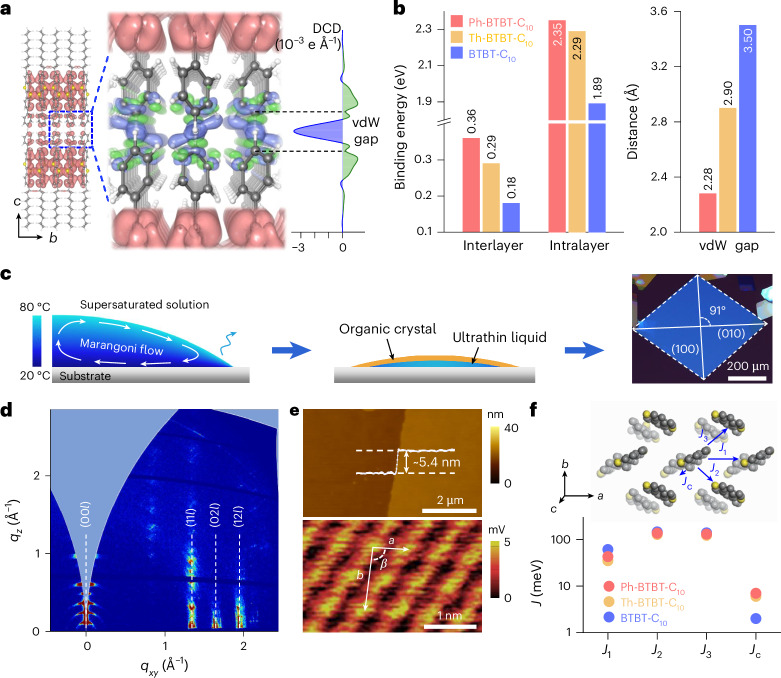
Conjugated phenyl pairs in Ph-BTBT-C_10_ bilayers. **a**, Schematic of HTH Ph-BTBT-C_10_ with a zoomed-in view of phenyl pairs and the visualized in-plane orbital overlap in the BTBT layers in a three-dimensional view. Right: interlayer DCD at the phenyl pairs. Pink, green and blue colours represent the total charge density, charge accumulation and reduction in DCD, respectively. **b**, Interlayer and intralayer binding energies and vdW gap in Ph-BTBT-C_10_, Th-BTBT-C_10_ and BTBT-C_10_. **c**, Illustration of the supersaturated crystallization process: Marangoni-flow-assisted crystal growth, long-term crystal relaxation and obtained cross-polarized optical microscopy image of a Ph-BTBT-C_10_ crystal. **d**, GIWAXS patterns of the HTH Ph-BTBT-C_10_ crystals with distinct diffraction spots. *q*_*xy*_ and *q*_*z*_ represent the in-plane and out-of-plane scattering vectors. **e**, AFM and high-resolution AFM images of the HTH Ph-BTBT-C_10_ films, displaying a bilayered step and highly ordered molecular packing. *a*, *b* and *β* are the in-plane lattice parameters. **f**, Illustration of Ph-BTBT-C_10_ molecular packing and calculated transfer integrals *J* of Ph-BTBT-C_10_, Th-BTBT-C_10_ and BTBT-C_10_.

In this Article, we report an examination of charge transport in an asymmetrically substituted derivative of benzothienobenzothiophene, 2-decyl-7-phenyl-[1]benzothieno[3,2-*b*][1]benzothiophene (Ph-BTBT-C_10_)^23,24^, in a regular organic FET with a conventional gate dielectric. Ph-BTBT-C_10_ is a liquid-crystalline molecule that can be induced to adopt a head-to-head (HTH) bilayer structure (Fig. 1a) in thin, solution-deposited films, which are essentially single crystalline over the length scale probed in FET devices. This overcomes thermal expansion mismatch that leads to mechanical cracks at low temperatures in temperature-dependent transport measurements on thicker molecular single crystals such as rubrene^11,25^. Our organic crystals exhibit metallic charge transport down to 8 K; the conductivity of the field-induced accumulation layer increases continuously with decreasing temperature and reaches values up to 245 S cm^−1^. The corresponding extracted field-effect mobility is band like and reaches values of >100 cm^2^ V^−1^ s^−1^ at 20 K. We examine the origin of these transport properties in terms of the electronic structure of HTH molecular bilayers, as well as demonstrate a method for inducing defect states in a controlled manner that allows a disorder-driven metal–insulator transition (MIT) to be observed in such systems.

## Conjugated molecular bilayers with vdW bridges

We first theoretically analyse the electronic structure of the Ph-BTBT-C_10_ bilayers, in which the phenyl pairs within the HTH molecules bridge the adjacent conjugated planes of BTBT layers (Fig. 1a). Density functional theory (DFT) calculations were used to analyse the electronic structure of ideal HTH Ph-BTBT-C_10_, particularly focusing on the region of phenyl pairs. For comparison, 2-decyl-7-thiophene-[1]benzothieno[3,2-*b*][1]benzothiophene (Th-BTBT-C_10_) and 2-decyl-[1]benzothieno[3,2-*b*][1]benzothiophene (BTBT-C_10_) with the same HTH packing were also examined. All materials exhibited strong intralayer orbital overlap between the BTBT molecular cores, with a nearly isotropic in-plane orbital conjugation (Supplementary Text 1 and Supplementary Fig. 1). Interestingly, when comparing the electronic states of Ph-BTBT-C_10_, Th-BTBT-C_10_ and BTBT-C_10_, there was noticeable wavefunction amplitude on the phenyl and thiophene pairs, and the out-of-plane interlayer tunnelling probability between the two BTBT layers could be much enhanced within the bilayer across these pairs. In Ph-BTBT-C_10_, the states at the valence band maxima (Supplementary Fig. 1d, VB1–4) contributed to the in-plane conducting channels. The differential charge density (DCD) is defined as the difference between the charge density of the bilayer and that calculated for the top and bottom layers in the absence of the other one and, thus, represents the charge redistribution among bilayer formation. The DCD plot for Ph-BTBT-C_10_ (Fig. 1a) displays an absolute value of the maximum density up to 3.51 × 10^−3^ e Å^−1^ at the gap of the phenyl pair, which is ~49% higher than that of the thiophene pair (2.36 × 10^−3^ e Å^−1^), whereas both molecules markedly exceed that of BTBT-C_10_ (0.17 × 10^−3^ e Å^−1^; Supplementary Fig. 2 and Supplementary Table 1). These results indicate that the interlayer vdW interactions in between HTH bilayers can be enhanced in Ph-BTBT-C_10_ and Th-BTBT-C_10_.

The vdW binding energies (*E*_b_) for the bilayers of all three materials were further calculated (Fig. 1b (left) and Supplementary Fig. 2). The interlayer *E*_b_ per Ph-BTBT-C_10_ molecular pair was 0.36 eV; it was larger than that in Th-BTBT-C_10_ (0.29 eV), and double that in BTBT-C_10_ (0.18 eV). In Ph-BTBT-C_10_ and Th-BTBT-C_10_, the intralayer *E*_b_ for in-plane adjacent molecules was also enhanced by more than 20% from BTBT-C_10_. The enhanced inter- and intralayer *E*_b_ in molecular semiconductors suggest an increased structural rigidity, which can lead to a suppression of carrier scattering by molecular vibrations through the deformation potential mechanism^26,27^. Besides, the calculated vdW gap between the phenyl rings of a Ph-BTBT-C_10_ pair was only 2.28 Å, which was much narrower than Th-BTBT-C_10_ (2.90 Å) and BTBT-C_10_ (3.50 Å; Fig. 1b (right) and Supplementary Fig. 2a,c,e). A detailed study of the role of dynamic disorder in the molecular bilayers goes beyond the scope of the present study, but in an attempt to assess the effect of phenyl pairs on the structural dynamics, we compared the Raman spectra between the HTH crystalline bilayers and the head-to-tail smectic E (SmE) phase of Ph-BTBT-C_10_. The phenyl pairs are absent in the SmE phase^28^. Generally, molecular vibrations can lead to transfer integral (*J*) fluctuations (Δ*J*/*J*), which is also known as the strength of dynamic disorder^8^. It has been demonstrated that a simple way to assess the role of such thermal fluctuations is by analysing the Raman spectra by determining the parameter *R*, which is defined as the integrated intensity ratio between the low-frequency Raman modes and frequency-divided high-frequency modes^29,30^. These measurements were performed on powder samples to eliminate the effects of molecular orientation with respect to the incident laser beam. Ph-BTBT-C_10_ in its crystalline phase exhibits a lower *R* value than in its SmE phase (Supplementary Text 2 and Supplementary Fig. 3). This indicates that the presence of phenyl pairs could indeed reduce the amplitude of molecular vibrations and the ensuing dynamic disorder, favouring efficient charge transport. The importance of reduced dynamic disorder in accounting for the excellent charge transport properties of Ph-BTBT-C_10_ will need to be investigated in more detail in a future study using techniques such as diffuse electron scattering^31,32^. Whether reduced dynamic disorder is relevant or not, our simulations reported here suggest that one of the key factors in the excellent charge transport properties is likely to be that the phenyl pairs facilitate favourable charge transfer by tunnelling through the vdW gap to the adjacent conjugated BTBT plane, such that charge transport is essentially distributed across the bilayer.

To investigate the charge transport in Ph-BTBT-C_10_ single crystals experimentally, we developed a solution-deposition method for growing ultrathin (two bilayers) single-crystal films. A supersaturated solution in a high-boiling-point solvent was drop cast onto a hydrophilic SiO_2_ substrate (Fig. 1c, Supplementary Text 3 and Supplementary Figs. 4–7). Temperature-gradient-induced Marangoni flow spontaneously formed with an inward direction at the solution–substrate interface^33,34^, facilitating the initial growth of thin Ph-BTBT-C_10_ crystals (Fig. 1c, left). Several minutes after the drop casting, the organic crystals with a size of several hundred micrometres were observed floating on top of an ultrathin liquid film underneath (Supplementary Figs. 5 and 6). The residual liquid evaporated fairly slowly, and over a period of approximately 2 h, the crystals slowly grew on top of the liquid film before all the liquid had evaporated (Supplementary Figs. 4 and 5). We assumed that such slow growth on top of a liquid film was beneficial for obtaining high-quality single-crystal films with a low density of defects. Eventually, flat rhombus-shaped ultrathin crystals with a large size over 600 μm were formed on the substrate (Fig. 1c, right). The crystals exhibited uniform brightness changes under crossed polarizers with the polarization angle (Supplementary Fig. 8). The measured angle between the (100) and (010) planes in our crystals was ~91°, which was very close to that in ideal Ph-BTBT-C_10_ single crystals^24^.

Figure 1d shows the diffraction patterns of a crystal measured by grazing-incidence wide-angle X-ray scattering (GIWAXS), indicating a highly crystalline structure. Despite the ultrathin film thickness, at least five orders of (00*l*) diffractions can be seen due to the interlayer stacking in the out-of-plane direction. The atomic force microscopy (AFM) image shows very smooth molecular terraces separated by bilayered surface steps with a height of ~5.4 nm, which is double the length of one Ph-BTBT-C_10_ molecule (Fig. 1e). A high-resolution AFM image is able to resolve the molecular packing without apparent defects or distortion. These structural characterizations clearly show that our films exhibit a good agreement with theoretical values of ideal HTH Ph-BTBT-C_10_ single crystals^24^ (Supplementary Fig. 9). In particular, in its SmE phase, Ph-BTBT-C_10_ molecules arrange in a head-to-tail interpenetrating interlayer packing, possessing a *c*-axis lattice parameter of ~2.7 nm, which is contradictory to that of our prepared single crystals^23^ (Supplementary Fig. 10).

The molecular packing of Ph-BTBT-C_10_ is shown in Fig. 1f, together with the relevant in-plane and out-of-plane transfer integrals. We carried out DFT calculations and found that the in-plane transfer integrals were nearly isotropic, which facilitates a two-dimensional (2D) charge transport and high carrier mobilities in the conjugated BTBT layers^35^. Generally, out-of-plane transfer integrals (*J*_c_) are fairly negligible in layered dialkylated molecular semiconductors. For instance, a popular semiconducting small molecule, C_8_-BTBT-C_8_, has an ultralow *J*_c_ (~0.01 meV) across the long insulating alkyl side chains. Across the phenyl and thiophene pairs, Ph-BTBT-C_10_ and Th-BTBT-C_10_ yielded a remarkably large *J*_c_ of 7 and 6 meV in their HTH packing, respectively, promoting enhanced interlayer charge transfer, whereas in BTBT-C_10_, *J*_c_ was only 2 meV. Combined with the enhanced interlayer vdW interactions and high tunnelling probability across the phenyl or thiophene pairs, referred to as ‘vdW bridges’, both HTH Ph-BTBT-C_10_ and Th-BTBT-C_10_ crystals possess improved out-of-plane interlayer charge transfer that links the in-plane 2D transport networks of adjacent BTBT layers. Our hypothesis is that the presence of such strong vdW bridges could be beneficial for charge transport, which would suggest that Ph-BTBT-C_10_ should outperform Th-BTBT-C_10_; both molecules should exhibit better transport properties than BTBT-C_10_, which lacks such vdW bridges.

## Observation of metallic charge transport

**Fig. 2:**
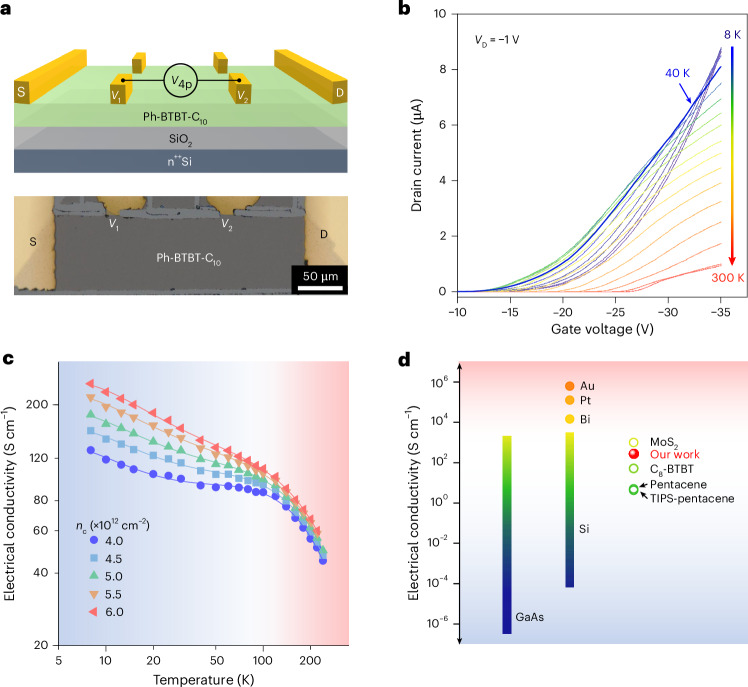
Metallic charge transport in Ph-BTBT-C_10_ bilayers. **a**, Device structure with a four-point-probe architecture (top) and the corresponding optical image (bottom). S, D, *V*_1_, *V*_2_ and *V*_4p_ represent the source electrode, drain electrode, the voltages at the inner two probes and corresponding potential drop, respectively. The dielectric is 50-nm-thick SiO_2_. **b**, Temperature-dependent linear transfer characteristics at a drain voltage *V*_D_ of −1 V. **c**, Temperature-dependent four-point-probe electrical conductivity *σ*_4p_ at various carrier concentrations *n*_c_, all of which exhibit metallic behaviour. **d**, Conceptual colour bar of the electrical conductivities for various materials, including undoped organic semiconductors, inorganic semiconductors and metals. All the organic semiconductors are single crystalline, except TIPS-pentacene, which is polycrystalline.

To investigate whether the vdW bridges benefit the charge transport properties, we performed top-contact, bottom-gate FET measurements on single crystals of Ph-BTBT-C_10_ with a thickness of 10.4 nm (Supplementary Fig. 9d); a gated four-point-probe configuration was used to exclude the effects of contact resistance (Fig. 2a, Supplementary Text 4 and Supplementary Figs. 11 and 12). The measured distance between the voltage probes was Δ*L* = 130 μm, and a channel width of *W* = 80 μm was defined by mechanically patterning the film; the gate dielectric capacitance was measured to be *C*_i_ = 63.8 nF cm^−2^ (Supplementary Fig. 13), which is in good agreement with the expected capacitance of a 50-nm-thick SiO_2_ gate dielectric. Figure 2b shows the typical temperature-dependent transfer characteristics in the linear regime measured from 8 K up to 300 K at a drain voltage (*V*_D_) of −1 V. Remarkably, at a sufficiently high gate voltage (|*V*_G_ | > 34.5 V), the drain current increases continuously with decreasing temperature from room temperature all the way down to 8 K. Similarly, the four-point-probe-extracted sheet conductivity, *σ*_4p_, determined at various *n*_c_ values in the bilayered Ph-BTBT-C_10_ crystals also exhibited a metallic temperature dependence (*∂σ*_4p_/*∂T* < 0) across the entire temperature range (Fig. 2c). This is remarkable, since metallic charge transport tends to be more difficult to observe than the band-like transport that has been reported in some organic FETs over a limited temperature range, because, especially at cryogenic temperatures, some of the applied *V*_G_ is needed to fill up deep trap states induced by residual disorder, resulting in an increase in *V*_T_ and decrease in *n*_c_ (Supplementary Text 5 and Supplementary Table 2). At 8 K, the value of *σ*_4p_ reached an ultrahigh value of 245 S cm^−1^ at *V*_G_ = −35 V and *V*_D_ = −1 V. For the calculation of *σ*_4p_, we assumed that the charge transport was confined to the first HTH bilayer at the interface with a thickness of 5 nm. The mean free path (*l*_e_) of the charge carriers was estimated using *l*_e_ = ℏ*k*_F_*G*/(*e*^2^*n*_c_), where *k*_F_ = (2π*n*_c_)^1/2^ is the Fermi wavevector, ℏ is the reduced Planck constant and *G* is the sheet conductance^18^. With the values experimentally obtained at 8 K (*G* are 65 and 122 μS at *n*_c_ = 4.0 and 6.0 × 10^12 ^cm^−2^, respectively), *l*_e_ for the hole carriers in HTH Ph-BTBT-C_10_ are ~3.4 nm and ~5.1 nm, corresponding to five- and eight-unit cells of Ph-BTBT-C_10_, respectively (Supplementary Text 6). This large *l*_e_, which is beyond the intermolecular spacing, is consistent with the observation of metallic charge transport in Ph-BTBT-C_10_. We compared the high electrical conductivity of Ph-BTBT-C_10_ of 245 S cm^−1^ at 8 K with those of other undoped organic and inorganic semiconductors^12,22,35^ (Fig. 2d and Supplementary Fig. 14; note that all the conductivity values quoted for different materials were not measured at 8 K, but at the temperature at which these materials exhibited their highest conductivities; see Supplementary Table 2 for more details). It was higher than all other organic semiconductor FETs reported in the literature, and comparable with heavily doped silicon and high-quality wide-bandgap GaAs bulk^36,37^, even approaching that of MoS_2_ (ref. ^38^).

**Fig. 3:**
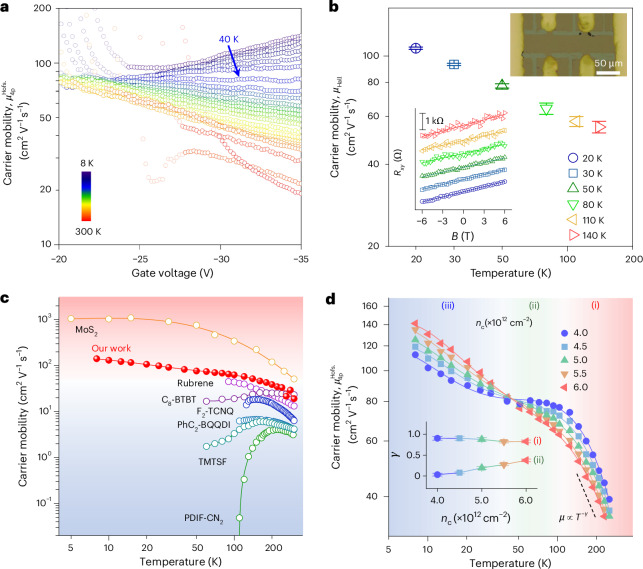
Charge carrier mobility of Ph-BTBT-C_10_ bilayers. **a**, Gate-voltage-dependent Hofstein mobility *μ*^Hofs.^_4p_ at various temperatures. Some of the data points near the threshold voltage are shaded to indicate that the mobility extraction method ceases to be valid close to the threshold. **b**, Temperature dependence of Hall mobility *μ*_Hall_. The top inset displays the optical image of the Hall device. The bottom inset shows the Hall resistance *R*_*xy*_ of Ph-BTBT-C_10_ at various temperatures (points) and linear fitting (lines) at gate voltage *V*_G_ of −110 V. The dielectric is 200-nm-thick SiO_2_. The Hall coefficient *R*_H_ was the slope of the *R*_*xy*_−*B* curve (*B* is the magnetic field), and the longitudinal conductance was calculated using the four-point-probe voltage drop recorded simultaneously. The error bars in *μ*_Hall_ originate from the uncertainty in *R*_*xy*_ from fitting and represent one standard deviation. **c**, Summary of the carrier mobilities in high-mobility single-crystal organic semiconductors and MoS_2_ with a band-like signature. The shaded regions are separated by a carrier mobility of 50 cm^2^ V^−1^ s^−1^. **d**, Temperature-dependent *μ*^Hofs.^_4p_ at various *n*_c_ values. The dashed line represents the power-law dependence of *μ* ∝ *T*^−*γ*^ (*γ* is the power-law exponent), and the inset shows *γ* fitted in regimes (i) and (ii). The error bars in *γ* originate from the power-law-fitting uncertainty and represent one standard deviation. The blue-, green- and red-shaded regions represent temperature ranges of ~8–40 K, ~40–100 K and ~100–280 K, respectively.

The conclusion that metallic charge transport is achievable in bilayered Ph-BTBT-C_10_ FETs across the entire temperature range is unambiguous and follows directly from the measurement of the monotonic negative temperature dependence of *σ*_4p_. We were also interested in extracting the corresponding charge carrier mobility values. This needs to be done with care: although the transfer characteristics in Ph-BTBT-C_10_ FETs measured in the linear-operation regime exhibited only slight hysteresis that remained nearly unchanged throughout the temperature range (Supplementary Fig. 15), which is evidence for good device stability, they exhibit pronounced nonlinearities (Fig. 2b). This nonlinearity is unlikely to be an artefact due to the contact resistance, as our four-point-probe method took the contact resistance effects into account, but instead is likely to reflect a true dependence of the field-effect mobility on gate voltage. At low temperatures below 40 K, the characteristics are superlinear, whereas at higher temperatures, they become sublinear at a higher gate voltage, that is, the derivative of the transfer characteristics was reduced at the highest gate voltages. At only around 40 K, the current rises linearly with the gate voltage above *V*_T_. When measuring devices near room temperature, we also observe a strong *V*_T_ shift towards a higher gate voltage; this will be discussed in more detail below. To minimize the potential for mobility extraction artefacts under strong *V*_G_ dependence, the mobility as a function of gate voltage was extracted using the drain current integration method proposed by Hofstein in 1963 (*μ*^Hofs.^_4p_; Methods)^39^ rather than using the direct derivative of the four-point-probe-measured transfer characteristics in the linear regime (*μ*^deri.^_4p_; Fig. 3a and Supplementary Text 7). Near 40 K, the transfer characteristics are the most ideal and *μ*^Hofs.^_4p_ reaches a high, nearly gate-voltage-independent value of 80 cm^2 ^V^−1^ s^−1^. At 8 K and the highest gate voltage, the extracted *μ*^Hofs.^_4p_ values reach even higher values of 141 cm^2 ^V^−1^ s^−1^. In addition, *μ*^Hofs.^_4p_ increases obviously with the decreasing temperature within the entire temperature range, indicating a band-like charge transport in Ph-BTBT-C_10_. Another approach to analyse the robustness of the extracted mobility values is to use the so-called reliability factor (*r*), which has been suggested for benchmarking the performance of non-ideal FETs to that of an ideal device with an effective mobility (*μ*_eff_ = *r* × *μ*^deri.^_4p_)^40^. Therefore, we used this method and found that a band-like dependence was also displayed across the entire temperature range with still high *μ*_eff_, indicating that the band-like charge transport was robust and was not dependent on the method for extracting mobility (Supplementary Text 4 and Supplementary Fig. 16).

To further validate the high mobility values, Hall effect measurements were also performed at 50 K, near the point at which the transfer characteristics exhibit a near-linear gate voltage dependence (Supplementary Fig. 17a). The carrier concentration (*n*_Hall_) of 4.2 × 10^12 ^cm^−2^ extracted from the measured Hall resistance is consistent with that expected from the gate dielectric capacitance. A Hall mobility of 85 cm^2^ V^−1^ s^−1^ at 50 K was obtained, which is close to the value of *μ*^Hofs.^_4p_ extracted at this temperature. We also plotted the Hall mobility as a function of temperature (Fig. 3b). For these measurements, we minimized the bias and current stress effects occurring in conventional magnetic-field-sweeping Hall measurements at a constant current by measuring transfer scans at changing magnetic fields to extract Hall mobilities at various temperatures (Supplementary Fig. 17b,c). High-value Hall mobilities close to *μ*^Hofs.^_4p_ and a negative temperature coefficient were obtained, reaching a Hall mobility of ~108 cm^2^ V^−1^ s^−1^ at 20 K.

Therefore, we can conclude that the phenyl vdW bridges in Ph-BTBT-C_10_, which lead to an enhanced out-of-plane charge transfer and suppressed molecular vibrations, are potentially a key enabling factor for the intrinsic metallic charge transport with a record-high electrical conductivity, a band-like temperature dependence of mobility with values of >100 cm^2^ V^−1^ s^−1^ at low temperatures and a long charge delocalization length. We performed repeated gate voltage sweeps of this Ph-BTBT-C_10_ sample, and further examined the charge transport behaviour for another two samples (Supplementary Figs. 18 and 19). All samples maintained a metallic charge transport down to 20 K, and both electrical conductivity and carrier mobility are comparable with the sample shown in Fig. 2. We also performed similar measurements on Th-BTBT-C_10_ and BTBT-C_10_. In these molecules, below 160 K and 260 K, *σ*_4p_ rose with an increase in temperature, presenting lower maximum values of 27 and 14 S cm^−1^, respectively. Maximum *μ*^Hofs.^_4p_ values were 12 and 8 cm^2^ V^−1^ s^−1^, and *μ*_eff_ calculated using the *r* factor were 8 and 3 cm^2^ V^−1^ s^−1^, respectively (Supplementary Fig. 20). Although these two molecules clearly exhibit less-ideal charge transport properties (which may at least partially be due to a less extensive optimization), there is clear evidence that Th-BTBT-C_10_ exhibits superior low-temperature transport to BTBT-C_10_. This is consistent with our above hypothesis that vdW bridges can be beneficial for charge transport. To provide a proof with general validity, a wider range of molecular crystals with and without vdW bridges should be investigated in the future.

The mobility values observed in our Ph-BTBT-C_10_ FETs at low temperatures are higher than reported in a previous, careful transport study on Ph-BTBT-C_10_ (ref. ^19^; Supplementary Text 8 and Supplementary Fig. 21). We speculate that this can be attributed to differences in materials processing and device architecture, including the use of our slow, supersaturated crystallization method as opposed to the blade coating and careful optimization of the evaporation process for the top source–drain contacts, which yielded robust electrodes that maintained low contact resistance down to low temperatures (Supplementary Texts 3 and 9 and Supplementary Fig. 22). Furthermore, our measurement protocol started the electrical measurements at low temperatures to ensure a clean platform for charge transport and avoided measurements at high temperatures before measuring the low-temperature characteristics (Supplementary Text 8). The latter aspect is discussed in more detail below.

We benchmarked our temperature-dependent mobility with that of other reported high-performance organic semiconductors^20,35,41–44^ (Fig. 3c and Supplementary Table 2). Our obtained mobility not only exceeded that of other organic semiconductors nearly within the whole temperature range but also displayed a band-like transport across the widest temperature range without suffering a crossover into a thermally activated regime at the lowest temperatures. For instance, rubrene possesses a band-like transport only down to 90 K with a maximum mobility of ~45 cm^2^ V^−1^ s^−1^ (ref. ^20^). For comparison, we also included the data for MoS_2_, one of the most promising 2D atomic semiconductors, which requires high film quality and careful interface control for observing such a wide-temperature-range band transport^45^.

In semiconductors with an ideal lattice, such as silicon, the lattice vibrations are the only scattering process leading to charge mobility to continuously increase with decreasing temperature, enabling the observation of band transport. However, when introducing even a low level of doping (~10^14^ cm^−3^) in single-crystal silicon, this band transport can hardly be maintained, and a crossover is observed at ~30 K, below which the mobility reduces with decreasing temperature^46^. Given the lack of any rigorous purification in our sample fabrication protocol, this makes the observation of band-like transport in our Ph-BTBT-C_10_ single crystals down to 8 K even more remarkable.

To better understand the charge transport behaviour in HTH Ph-BTBT-C_10_, the temperature dependencies of *μ*^Hofs.^_4p_ are replotted in Fig. 3d at various *n*_c_ values. In regime (i) (120–240 K), *μ*^Hofs.^_4p_ is well fitted by a power-law dependence (*μ*^Hofs.^_4p_ ∝ *T*^−*γ*^) at all *n*_c_ values, yielding a nearly identical exponent *γ* ranging from 0.83 to 0.91, which is in good agreement with the theoretical values for organic semiconductors with isotropic transfer integrals predicted within the transient localization framework^47^. In this regime, the charge mobility decreases when *n*_c_ increases, corresponding to the sublinear behaviour in the transfer curves shown in Fig. 2b discussed above. This can, in principle, be attributed to the effect of charges becoming more closely attracted to the interface with the SiO_2_ gate dielectric at high gate voltages and experiencing stronger interface scattering/interactions with dipolar disorder in the dielectric. However, in our system, the charge carriers are separated from the dielectric surface by a layer of long alkyl chains and we, therefore, propose an alternative explanation in terms of Coulomb interactions among carriers. In molecular systems with a low degree of structural and energetic disorder, and charges confined to a 2D mono- or bilayer at the interfaces, the Coulomb interactions can contribute to the barrier height experienced during charge transfer, which was recently investigated by some of us for C_8_-BTBT-C_8_ single-crystal films^48^ (Supplementary Fig. 24). Potentially, a large gate field could lead to a charge redistribution within the molecular bilayers and increase the charge concentration in the Ph-BTBT-C_10_ layer closest to the interface, resulting in the observed reduction in mobility. In regime (ii) (40–120 K), as the temperature further decreases, the charge mobility for *n*_c_ = 4.0 × 10^12^ cm^−2^ remains nearly unchanged (from 80 cm^2^ V^−1^ s^−1^ at 40 K to 74 cm^2^ V^−1^ s^−1^ at 120 K). At the crossover point between regime (ii) and (iii) (40 K), the mobility is independent of *n*_c_, which corresponds to the ideally linear transfer curve shown in Fig. 2b. From 40 K to 8 K (regime (iii)), the mobility rises with increasing *n*_c_ (the superlinear transfer curves shown in Fig. 2b). *V*_G_ dependence in regime (iii) is typical of the behaviour of many organic semiconductors, and most likely reflects the filling of residual shallow trap states at the interface between the organic semiconductor and the gate dielectric^22^.

Our analysis reveals that the ultrahigh charge carrier mobilities and metallic transport in Ph-BTBT-C_10_ single crystals can be understood in terms of the enhanced interlayer interactions across the phenyl vdW bridges. The existence of these vdW bridges potentially leads to a suppression of molecular vibrations and a redistribution of charge density across the bilayers, which helps form a transport network that involves both molecular layers that make up the bilayer closest to the interface. In this way, the effects of Coulomb interactions among carriers or interfacial disorder can be minimized, leading to a wide-temperature-range metallic charge transport. Despite having evaluated only three molecules in this work, our results suggest a potential role of vdW bridges in benefitting charge transport, which may inspire the research community to explore this class of molecules more widely. At present, Ph-BTBT-C_10_ remains the system in which these excellent metallic transport properties can be observed most cleanly. Given that with any new organic semiconductor material, large effort over multiple years of research is usually required to improve the material’s purity and to optimize the fabrication conditions; therefore, this is not surprising. However, we believe that there is evidence to justify a more concerted future research effort to fully replicate the transport properties of Ph-BTBT-C_10_ in other molecular semiconductors with phenyl bridges.

## Disorder-driven MIT

We now turn to a discussion of the observation in Fig. 2b that *V*_T_ increased markedly when measuring the devices close to room temperature. This was a feature of our evaporated source–drain electrodes, which resulted in a relatively low *μ*_eff_ of 5.1 cm^2^ V^−1^ s^−1^ at room temperature, consistent with the value reported in ref. ^19^ using evaporated electrodes (Supplementary Fig. 21b). We further found that devices with evaporated electrodes exhibited a marked increase in contact resistance (*R*_c_) near room temperature, maintaining low *R*_c_ values at low temperatures (Supplementary Text 9 and Supplementary Fig. 22). This is attributed to the field-induced and temperature-activated electromigration of metal ions from evaporated gold contacts into molecular crystals, which has been observed directly in previous studies on BTBT and was found to be suppressed below 200 K (ref. ^49^). This explains why we were able to achieve a low *V*_T_ and a low *R*_c_ at low temperatures by simply avoiding to measure the device above 200 K before cooling down. By using mechanically transferred, laminated electrodes onto our single crystals, we were able to achieve a low *R*_c_ at room temperature, along with a small *V*_T_, a high *r* factor of 90% and an improved *μ*_eff_ of 14.0 cm^2^ V^−1^ s^−1^. These results were only slightly lower than the high-performance devices reported in ref. ^50^ using the same electrode process (*r* factor of 92% and *μ*_eff_ of 18.4 cm^2^ V^−1^ s^−1^; Supplementary Text 8). However, we could not use devices with laminated contacts for the low-temperature measurements, as they were found to be insufficiently robust when cooling the devices to low temperatures and their *R*_c_ increased markedly at low temperatures (Supplementary Text 9 and Supplementary Fig. 22). This is attributed to the general trade-off between the need to induce some interfacial roughness to promote adhesion and the need to minimize interfacial roughness to suppress electromigration. Moreover, in the temperature-dependent photoluminescence (PL) spectra of the sample that was electrically measured at room temperature, we observed an intensified 0–0 transition that should be forbidden in ideal Ph-BTBT-C_10_ (Supplementary Text 10 and Supplementary Fig. 25). Thus, the increased *V*_T_ near room temperature can also be attributed to an electric-field-induced generation of defect states at high temperatures^51^. This was further confirmed by cyclical electrical measurements, where *V*_T_ increased during the warming-up process (from 200 K to 300 K) and remained a high value during the cooling-down process (from 300 K to 100 K; Supplementary Text 11 and Supplementary Fig. 26). Therefore, these results indicated a persistent existence of disorder once introduced by applying voltages to the device at high temperature (>200 K). Despite the existence of disorder, we can still obtain a negative *μ*^Hofs.^_4p_−*T* dependence, which reflected that the band-like behaviour was dominant in charge transport and that the overall quality of our crystals was high. Nevertheless, it also allowed us to introduce such ‘stable’ disorder into the films in a controlled manner, by carrying out a stress measurement at a specific gate voltage in the ‘ON’ state for 240 s, and then study the effects on the temperature dependence of charge transport during a cooling-down process. Using this approach, a disorder-driven MIT can be observed, which is rarely reported in organic semiconductors^52^.

**Fig. 4:**
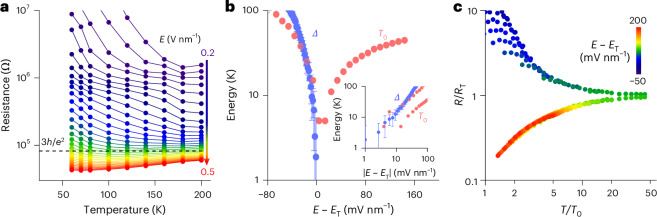
Disorder-driven MIT and quantum critical scaling in Ph-BTBT-C_10_ bilayers. **a**, Temperature dependence of sheet resistance under varying electric fields. *h* and *e* represent the Planck constant and elementary charge, respectively. **b**, Continuously vanishing activation gap *Δ* and temperature scaling parameter *T*_0_ as the electric field *E* approaches the critical field *E*_T_. Both parameters follow a power-law dependence on |*E* − *E*_T_| with similar exponents. The error bars originate from the fitting uncertainty in *R*−*T* dependence and represent one standard deviation. **c**, Temperature-dependent sheet resistance *R* curves near the MIT collapse onto two branches. The resistance curves are scaled by that at the critical field *R*_T_(*T*); the temperatures are scaled by field-dependent *T*_0_.

The temperature dependence of sheet resistance (*R*) of Ph-BTBT-C_10_ with disorder introduced by stressing the device at 200 K showed a typical MIT from a metallic regime at a high gate voltage to a thermally activated regime at a low gate voltage with a crossover at the critical field (*E*_T_) of 0.356 V nm^−1^ (Fig. 4a, Supplementary Text 12 and Supplementary Fig. 27). The critical resistance (*R*_T_), which represented the transition between metallic and insulating phases, was calculated to be ~80 kΩ (corresponding to ~3*h*/*e*^2^, where *h* is the Planck constant). This value was within the typical range for semiconductors, such as silicon^53^. In the thermally activated regime (*E* < *E*_T_), the activation gap (*Δ*) for charge transport displayed a monotonic decrease as *E* approached *E*_T_, following a power-law dependence *Δ* ≈ |*E* − *E*_T_|^*vz*^, where *vz* = 1.08 (Fig. 4b). We further demonstrated the scaling collapse of the resistance curves near MIT^54^ (Fig. 4c). The value of *R* was normalized by *R*_T_ at *E*_T_. The resistance curves near MIT symmetrically collapsed onto two branches after the temperatures were scaled by the field-dependent *T*_0_, following a scaling equation of *R*(*n*, *T*) = *R*_T_(*T*)*f*(*T*/*T*_0_). The top and bottom branches represented the insulating and metallic transport behaviours, respectively; they displayed a reflection symmetry about *R*/*R*_T_ = 1 in the log−log plot. Similar to *Δ*, *T*_0_ followed a power-law dependence *T*_0_ ≈ |*E* − *E*_T_|^*vz*^ with *vz* = 0.98 (Fig. 4b, inset). The field-temperature phase diagram for |lg(*R*/*R*_T_)| revealed a ‘fan-shaped’ structure that was widely observed for MIT^54^ (Supplementary Fig. 27c). We witnessed similar MIT in our sample with higher disorder densities; both critical gate voltage at which MIT occurred and sheet resistance increased with higher disorder densities (Supplementary Fig. 28). Therefore, our crystals with suppressed Coulomb interactions enabled the disorder-driven MIT, opening up possibilities in exploring physics for organic Mott−Anderson systems and in designing devices such as temperature-immune FETs (Supplementary Fig. 29), memory and optical modulators^55–57^.

## Conclusions

We have reported metallic charge transport down to 8 K in Ph-BTBT-C_10_ bilayer crystals with an electrical conductivity of up to 245 S cm^−1^, as well as charge carrier mobility values of more than 100 cm^2^ V^−1^ s^−1^ at 20 K. We attribute this to the phenyl pairs that bridge the conjugated BTBT planes, thereby suppressing the molecular vibrations and boosting the out-of-plane charge transfer to form a transport network beyond two dimensions. Furthermore, we demonstrated disorder-induced MIT in organic systems via the controlled introduction of disorder.

## Methods

### Sample preparation

Silicon substrates with 50-nm oxide layer (WaferPro) were cleaned by sonication in acetone and isopropanol for 10 min each. Ph-BTBT-C_10_ was purchased from Tokyo Chemical Industry. Ph-BTBT-C_10_ was dissolved in anisole with a concentration of 3 mg ml^−1^. The solution was heated at 80 °C for 10 min to ensure Ph-BTBT-C_10_ was completely dissolved. A droplet of Ph-BTBT-C_10_ solution was drop cast onto the substrate, and then it was naturally cooled to achieve a slow crystallization rate, which is critical for the growth of high-quality crystalline films (substrate temperature, ~20 °C). SmE Ph-BTBT-C_10_ samples were obtained by heating the crystalline Ph-BTBT-C_10_ at 150 °C for 10 min.

### Device fabrication and electrical characterizations

Ph-BTBT-C_10_ transistors were fabricated using a defined shadow mask with a channel length of 300 μm. The distance between the voltage-sensing electrodes (*V*_1_ and *V*_2_) was 130 μm. Here 40-nm-thick Au electrodes were thermally evaporated on the two-bilayer-thick Ph-BTBT-C_10_ crystals at a rate of ~0.03 Å s^−1^ for the initial 5 nm and ~0.3 Å s^−1^ for the following 35 nm. The ultralow evaporation rate for the initial 5 nm can confine the Au diffusion within the one-bilayer-thick region (Supplementary Fig. 22c). After Au evaporation, the Ph-BTBT-C_10_ films were further patterned mechanically with an electrical probe tip to define the channels with a typical width of 80 μm. Electrical characterizations of the Ph-BTBT-C_10_ transistors, including the measurements of transfer and output characteristics, were carried out using an Agilent B1500 semiconductor parameter analyser in the chamber of a Lake Shore vacuum probe station (~10^−5^ mbar), with a temperature range from 8 K to 300 K in a dark environment. For the observation of MIT, the device was measured in a cooling-down process. Hall effect measurements were performed in a He-gas-exchanged cryostat with a superconducting magnet (TeslatronPT, Oxford). The external field (*B*) was applied perpendicular to the substrate. Longitudinal voltage (*V*_*xx*_) and transverse voltage (*V*_H_) were recorded simultaneously by applying a constant d.c. current (*I* = 3 μA) and performing the transfer scans.

### Crystal structure characterizations

The surface morphology was analysed by AFM (Asylum Research MFP-3D) in the tapping mode. High-resolution AFM measurements were performed on an Asylum Cypher under ambient conditions with Asylum ARROW UHF AFM tips. GIWAXS measurements were performed at the beamline BL16B of the Shanghai Synchrotron Radiation Facility. The incident X-ray beam had a downward inclination of 0.367° after passing through the focusing mirror, a wavelength of 1.239 Å (photon energy, 10 keV) and a flux of ~2 × 10^11^ photons s^−1^. The sample was placed on a vibration isolation platform. GIWAXS data were collected during the dynamic compression process. X-ray diffraction measurements were performed using a Bruker D8 Advance diffractometer (Cu Kα radiation, 40 kV and 40 mA). Cross-sectional scanning transmission electron microscopy (STEM) specimen was prepared using a dual-beam focused-ion-beam system (Thermo Scientific Helios G4 UX). After that, bright-field/high-angle annular dark-field STEM images were acquired on an aberration-corrected STEM Titan^3^ G2 60-300 system with an accelerating voltage of 300 kV.

### Carrier mobility extraction

Because our FETs exhibit a nonlinear transfer characteristic indicating that the carrier mobility has a strong dependence on gate voltage, a robust method is needed to extract the gate-voltage-dependent carrier mobility. We used a method that goes back to the original derivation of the FET characteristics by Hofstein^39^ and applied this to our four-point-probe measurement configuration. In the gradual channel approximation, the channel current can be expressed as ${I}_{{\rm{D}}}={C}_{{\rm{i}}}{\mu }_{4{\rm{p}}}^{\mathrm{Hofs}.}W\times [{V}_{{\rm{G}}}-{V}_{{\rm{T}}}-V(z)]\frac{\Delta V}{\Delta z}\,$, where *z* denotes the position along the channel with potential *V*(*z*). An integration between the two voltage-sensing probes yields

$${I}_{{\rm{D}}}\mathop{\int }\limits^{{L}_{2}}_{{L}_{2}}{\rm{d}}z={C}_{i}{\mu }_{4{\rm{p}}}^{\mathrm{Hofs}{\rm{\cdot }}}W\mathop{\int }\limits^{{V}_{2}}_{{V}_{2}}\left[{V}_{{\rm{G}}}-{V}_{{\rm{T}}}-V\left(z\right)\right]{\rm{d}}V$$

$${I}_{{\rm{D}}}=\frac{{C}_{i}{\mu }_{4{\rm{p}}}^{\mathrm{Hofs}{\rm{\cdot }}}W}{\Delta {L}_{4{\rm{p}}}}\left[\left({V}_{{\rm{G}}}-{V}_{{\rm{T}}}\right)\left({V}_{2}-{V}_{1}\right)-\frac{1}{2}\left({V}_{2}^{2}-{V}_{1}^{2}\right)\right].$$

Thus, ${\mu }_{4{\rm{p}}}^{\mathrm{Hofs}{\rm{\cdot }}}=\frac{{I}_{{\rm{D}}}\Delta {L}_{4{\rm{p}}}}{{C}_{{\rm{i}}}W}\times \frac{1}{\left({V}_{{\rm{G}}}-{V}_{{\rm{T}}}\right)\left({V}_{2}-{V}_{1}\right)-\frac{1}{2}\left({V}_{2}^{2}-{V}_{1}^{2}\right)},$ where Δ*L*_4p_ is the distance between two voltage-sensing probes, and *V*_1_ and *V*_2_ represent the four-point-probe voltages. *V*_T_ is extracted using the second-derivative method^58,59^, which locates *V*_T_ at the maximum for the second derivative of the drain current with respect to the gate voltage $(\frac{{\rm{d}}{g}_{{\rm{m}}}}{{\rm{d}}{V}_{{\rm{G}}}})$ (Supplementary Text 13 and Supplementary Fig. 30).

This method is more accurate and robust than the commonly used derivative method, which calculates the gate-voltage-dependent mobility from ${\mu }_{4{\rm{p}}}^{\mathrm{deri}{\rm{\cdot }}}=\frac{\Delta {L}_{4{\rm{p}}}}{{C}_{{\rm{i}}}W}\times \frac{\partial \left[{I}_{{\rm{D}}}/\left({V}_{2}-{V}_{1}\right)\right]}{\partial {V}_{{\rm{G}}}}$. This is because the derivative method neglects a term $\propto \frac{\partial \mu }{\partial {V}_{{\rm{G}}}}$, which arises due to the product rule of differentiation when taking the derivative of the drain current. The method according to Hofstein is more robust because it is directly based on the definition of mobility relating the current to the carrier density and drift velocity. In Supplementary Fig. 16, we show the corresponding mobility values from the derivative methods to allow a direct comparison of the carrier mobility with those in refs. ^19,50^. Compared with the values extracted from the same measurement by the Hofstein method shown in Fig. 3a, the derivative method yields somewhat-higher overestimated values.

### PL and Raman measurements

For temperature-dependent steady-state PL and time-resolved PL measurements, an ytterbium-doped potassium gadolinium tungstate laser (PHAROS, LIGHT CONVERSION) was used as the excitation source. We used a frequency-tripled fundamental output at 343 nm. The samples were mounted under vacuum (<10^−3^ mbar) in a cold-finger liquid-helium cryostat (MicrostatHe, Oxford Instruments). A temperature controller (Mercury ITC, Oxford Instruments) monitored the temperature at a sensor mounted on the sample holder. PL signals were measured by a monochromator (Acton SP2500, Princeton Instruments) with a liquid-nitrogen-cooled silicon charge-coupled device (100B, PyLoN, Princeton Instruments). The time-resolved PL spectra were recorded using time-correlated single-photon counting (Time Tagger Ultra, Swabian Instruments) using an avalanche photodiode with a temporal resolution of ~50 ps (PicoQuant, MPD). Raman spectra were characterized with confocal laser Raman spectrometers (WITec alpha300, calibrated with silicon). The wavelength of the excitation laser was 488 nm and the power was 3 mW.

### DFT calculations

DFT calculations were performed using the generalized gradient approximation for the exchange–correlation potential, the projector augmented-wave method^60,61^ and a plane-wave basis set as implemented in the Vienna ab initio simulation package^62^. Dispersion correction was made at the vdW density functional level with the optB88 functional for the exchange potential. The kinetic energy cut-off for the plane-wave basis was set to 600 eV. A *k* mesh of 8 × 6 × 1 was adopted to sample the first Brillouin zone. The initial structure was built from the single-crystal data. The shape and volume of Ph-BTBT-C_10_ were fully relaxed until the residual force per atom was less than 0.02 eV Å^−1^. The optimized lattice constants of bulk Ph-BTBT-C_10_ are *a* = 5.92 Å, *b* = 7.30 Å and *c* = 52.84 Å. A bilayered structure was cleaved from the optimized bulk structure, and a vacuum layer of ~17 Å was added along the *c* direction. The shape and in-plane lattice of bilayer Ph-BTBT-C_10_ were also fully relaxed. The optimized bilayer Ph-BTBT-C_10_ possessed the lattice constants of *a* = 5.92 Å and *b* = 7.30 Å. Interlayer DCD was derived by *ρ*_DCD_ = *ρ*_total_ – *ρ*_top_ − *ρ*_bottom_. We calculated the charge densities of bare top layer (*ρ*_top_) and bottom layer (*ρ*_bottom_) using the same geometry and precision as those in the bilayered structure and subtracted them from the total charge density (*ρ*_total_). The charge transfer integral was calculated by *J* = (*E*_HOMO_ − *E*_HOMO−1_)/2, where *E*_HOMO_ and *E*_HOMO−1_ were energies of the two highest occupied molecular orbitals of a Ph-BTBT-C_10_ dimer. Th-BTBT-C_10_, BTBT-C_10_ and C_8_-BTBT-C_8_ were calculated using the same method as that for Ph-BTBT-C_10_ (refs. ^63,64^).

## Supplementary information

Supplementary Information Supplementary Texts 1–13, Figs. 1–30 and Tables 1–3.

## Data Availability

Source data that support the findings of this study are available from the corresponding authors upon reasonable request.
