# Supplementary material for: Metallic charge transport in conjugated molecular bilayers
Source: Nat Electron. 2026 Jan 20;9(3):246–56. doi: 10.1038/s41928-025-01553-5 (PMC13035466; doi:10.1038/s41928-025-01553-5)
Supplement: Supplementary file 1 — Supplementary Texts 1–13, Figs. 1–30 and Tables 1–3. [file 41928_2025_1553_MOESM1_ESM.pdf]

nature electronics

Supplementary information

https://doi.org/10.1038/s41928-025-01553-5

Metallic charge transport in conjugated molecular bilayers

In the format provided by the authors and unedited

Table of Contents

Supplementary Text 1. DFT calculations for Ph-BTBT-C_{10}, Th-BTBT-C_{10}, and BTBT-C_{10}. 4
Supplementary Text 2. Discussion on molecular vibrations. 5
Supplementary Text 3. Supersaturated crystallisation method. 6
Supplementary Text 4. Electrical characterisations for crystalline Ph-BTBT-C_{10} transistors........ 8
Supplementary Text 5. Metallic charge transport in organic field-effect transistors. 8
Supplementary Text 6. Delocalisation length. 10
Supplementary Text 7. Charge carrier mobility extraction and comprehensive description on OFET device performance. 10
Supplementary Text 8. Comparison of our Ph-BTBT-C_{10} device performance with that in references. 11
Supplementary Text 9. Devices using transferred electrodes and PVC-modified interfaces. 12
Supplementary Text 10. Photoluminescence spectroscopy. 13
Supplementary Text 11. Introduction of disorder in Ph-BTBT-C_{10} single crystals. 14
Supplementary Text 12. Observation of disorder-driven MIT. 15
Supplementary Text 13. Threshold voltage extraction in OFETs. 17
Supplementary Fig. 1 | Band structures and wavefunction norms of Ph-BTBT-C_{10}, Th-BTBT-C_{10}, and BTBT-C_{10} 18
Supplementary Fig. 2 | Differential charge densities (DCD) of Ph-BTBT-C_{10}, Th-BTBT-C_{10}, and BTBT-C_{10}. 19
Supplementary Fig. 3 | Raman spectra of crystalline and smectic E (SmE) Ph-BTBT-C_{10} powder samples. 20
Supplementary Fig. 4 | Illustration of the supersaturated crystallisation method and corresponding COMSOL simulations... 21
Supplementary Fig. 5 | Real-time Ph-BTBT-C_{10} crystal growth using the supersaturated crystallisation method. 22
Supplementary Fig. 6 | Real-time mapping of liquid bridge thickness and scanning transmission electron microscopy (STEM) images of Ph-BTBT-C_{10} crystal. 23
Supplementary Fig. 7 | Optimisations for Ph-BTBT-C_{10} crystal growth.................................... 24

Supplementary Fig. 8 | Cross-polarized optical microscopy images at various polarization angles. 25
Supplementary Fig. 9 | Summary of the lattice parameters and thicknesses of Ph-BTBT-C_{10} films. 26
Supplementary Fig. 10 | Film property and electrical characterisations for SmE Ph-BTBT-C_{10} films. 27
Supplementary Fig. 11 | Output curves for HTH Ph-BTBT-C_{10} OFET. 28
Supplementary Fig. 12 | Electrical characterisations for the Ph-BTBT-C_{10} OFET. 29
Supplementary Fig. 13 | Unit capacitance (C_i)-frequency (f) dependence for SiO_2 substrates used in our devices. 30
Supplementary Fig. 14 | Summary of the electrical conductance-temperature dependence in high-mobility organic semiconductors. 31
Supplementary Fig. 15 | Hysteresis in a HTH Ph-BTBT-C_{10} transistor. 32
Supplementary Fig. 16 | Carrier mobility extraction using direct derivative method. 33
Supplementary Fig. 17 | Temperature-dependent Hall measurements employing conventional magnetic-field-sweeping method and transfer-scanning method for the Ph-BTBT-C_{10} FET. 34
Supplementary Fig. 18 | Repeated gate sweeps of the Ph-BTBT-C_{10} sample in Fig. 2 in main text. 35
Supplementary Fig. 19 | Electrical characterisations for three Ph-BTBT-C_{10} samples. 36
Supplementary Fig. 20 | Electrical characterisations for crystalline Th-BTBT-C_{10} and BTBT-C_{10} transistors. 37
Supplementary Fig. 21 | Comparison of the electrical characteristics of Ph-BTBT-C_{10} transistors in ref. [15] and our work. 38
Supplementary Fig. 22 | Electrical characterisations for a Ph-BTBT-C_{10} transistor with transferred electrodes and corresponding electrode/semiconductor interface properties. 39
Supplementary Fig. 23 | Device performance of a Ph-BTBT-C_{10} transistor with PVC-modified semiconductor/dielectric interface. 40
Supplementary Fig. 24 | Hole-hole scattering-induced barrier height increase. 41
Supplementary Fig. 25 | Photoluminescence spectroscopy (PL) and time-resolved PL (TRPL) characterisations for Ph-BTBT-C_{10} films. 42

Supplementary Fig. 26 | Threshold voltage shift and corresponding transfer curves of a Ph-BTBT-C_{10} device. 43
Supplementary Fig. 27 | Phase diagram of Anderson-Hubbard model and temperature-dependent conductance of the Ph-BTBT-C_{10} with introduced disorder.......... 44
Supplementary Fig. 28 | Disorder-induced MIT in HTH Ph-BTBT-C_{10}. 45
Supplementary Fig. 29 | A temperature-immune OFET. 46
Supplementary Fig. 30 | Threshold voltage (V_T) extraction in OFETs. 47
Supplementary Table 1 | Calculated electronic structural parameters and transport parameters of Ph-BTBT-C_{10}, Th-BTBT-C_{10}, and BTBT-C_{10}. 48
Supplementary Table 2 | Summary of undoped high-mobility organic semiconductors and MoS_2 in literatures. 49
Supplementary Table 3 | Summary of threshold voltage in high-mobility OFETs. 50

Supplementary Text 1. DFT calculations for Ph-BTBT-C_{10}, Th-BTBT-C_{10}, and BTBT-C_{10}.

We first employed DFT calculations to study the band structures and electronic properties of Ph-BTBT-C_{10}, Th-BTBT-C_{10}, and BTBT-C_{10}. We found that adding phenyl or thiophene groups in the molecular structure induced small reductions in the band gap of Ph-BTBT-C_{10} (1.89 eV) and Th-BTBT-C_{10} (1.85 eV) when compared with BTBT-C_{10} (1.97 eV) (Supplementary Figs. 1a-c). Next, we visualized the wavefunction norms of valence band (VB) 1−4 states at G point for the three structures (Supplementary Figs. 1d-f). VB1&2 and VB3&4 states contributed to conducting channels in the *ab* plane[1]. Intermolecular wavefunction overlapping can be found in BTBT regions for the three molecular semiconductors. The electronic states in conjugated BTBT regions, specifically in the thiophene rings, served as the conducting channels in *ab* plane, similar to those in C_8-BTBT-C_8[1].

For Ph-BTBT-C_{10}, VB1&2 states were distributed over the top and bottom layers, whereas for Th-BTBT-C_{10} with thiophene pairs and BTBT-C_{10} without pairs, the states were predominantly distributed within one of the bilayers. These results suggested the strongest interlayer coupling in Ph-BTBT-C_{10}, which is analogous to the enhanced intermolecular interactions in 2,6-diphenylanthracene due to phenyl substitution[2]. Furthermore, VB3&4 states for the head-to-head (HTH) Ph-BTBT-C_{10} showed vertical wavefunction in the phenyl pairs. By contrast, no wavefunction overlap was found between neighboring layers of Th-BTBT-C_{10} and BTBT-C_{10} in all VB states. This feature allowed the van der Waals (vdW) phenyl pairs to serve as bridges that connect the adjacent BTBT planes, enhancing the out-of-plane interlayer tunnelling probability between the phenyl rings, which was further confirmed by the boosted interlayer transfer integrals and interlayer binding energies (Supplementary Table 1).

We analysed the interlayer DCD of Ph-BTBT-C_{10}, Th-BTBT-C_{10}, and BTBT-C_{10} in Supplementary Figs. 2a,c,e, which represent the charge redistribution on the molecules during the formation of HTH bilayer with respect to the isolated molecular layers (see DFT calculations in Methods). The formation of vdW phenyl pairs in Ph-BTBT-C_{10} and thiophene pairs in Th-BTBT-C_{10} led to a significant charge redistribution, which was much less pronounced in BTBT-C_{10}. Therein, at the midpoint of the interlayer region, as labelled by the black dashed line, the absolute values of DCD line profile along the out-of-plane direction were ~ 3.51×10^{-3} , 2.36×10^{-3}, and 0.17×10^{-3} e Å^{-1} for Ph-BTBT-C_{10}, Th-BTBT-C_{10}, and BTBT-C_{10}, respectively (Supplementary Figs. 2b,d,f). These results indicated the strongest interlayer vdW interactions in between the Ph-

BTBT-C_{10} layers and relatively strong interlayer vdW interactions in between the Th-BTBT-C_{10} layers compared to BTBT-C_{10}. Moreover, the strongest interlayer vdW interactions brought ~ 35% shorter vertical interlayer distance for Ph-BTBT-C_{10} (2.28 Å) and ~ 17% shorter vertical interlayer distance for Ph-BTBT-C_{10} (2.90 Å) compared to that of BTBT-C_{10} (3.50 Å). Generally, the interlayer charge exchange can be treated as tunnelling transmission, whose probability (P_{tu}) is highly dependent on the barrier width as $P_{tu} \sim \exp(-2k_F d)$, where k_F is the wave vector and d is the barrier width. Therefore, the tunnelling resistivity was markedly lower in Ph-BTBT-C_{10} with phenyl pairs compared with that in Th-BTBT-C_{10} and BTBT-C_{10}.

Supplementary Text 2. Discussion on molecular vibrations.

To assess the likely impact of the bilayer formation on the structural dynamics and molecular vibrations, we calculated the binding energies of the molecules within the bilayer, and measured the Raman spectra for the crystalline and SmE Ph-BTBT-C_{10} powder samples. As discussed in the main text, the charge transport is mainly dependent on the BTBT channel. The binding energies for Ph-BTBT-C_{10}, Th-BTBT-C_{10}, and BTBT-C_{10} bilayers are summarized in Supplementary Table 1. Ph-BTBT-C_{10} with vdW bridges exhibits the strongest inter- and intralayer binding energies, which induces the stiffest molecular packing, suggesting a suppression of molecular vibrations.

Besides, the charge carrier mobility is highly affected by the low-frequency vibrational modes[3,4]. Molecular vibrations can lead to the transfer integral fluctuations ($\Delta J/J$, which is known as the strength of dynamic disorder). $\Delta J/J$ can be estimated experimentally from the Raman spectra using the parameter R, which is the integrated intensity ratio between the low-frequency Raman modes and frequency-divided high-frequency modes. To investigate the effect of phenyl pairs on molecular vibrations, the HTH crystalline bilayers and the head-to-tail (HTT) smectic E (SmE) phase of Ph-BTBT-C_{10} were prepared for the Raman characterisations. The SmE sample was obtained by heating the purchased Ph-BTBT-C_{10} powder at 170 °C for ~ 30 min[5,6]. X-ray diffraction (XRD) patterns of the samples before and after the heating process confirmed the crystalline and SmE Ph-BTBT-C_{10} used in Raman measurements (Supplementary Figs. 3a,b). Then, the powder samples were further ground to eliminate effects of molecular orientation with respect to the incident laser beam. The obtained R value in crystalline Ph-BTBT-C_{10} (1520) sample was lower than that in the SmE one (1677) (Supplementary Fig. 3c). Therefore, our results are

indeed consistent with the hypothesis that the formation of phenyl pairs can suppress the molecular vibrations.

Supplementary Text 3. Supersaturated crystallisation method.

Based on the theoretical calculations of molecular orbitals, ideal HTH Ph-BTBT-C_{10} single crystals possessed a two-dimensional in-plane charge transport with isotropic transfer integrals. Thus, an equilibrium state of Ph-BTBT-C_{10} molecular packing was highly desirable in the sample preparation. Typical solution-processing strategies, such as spin-coating, dip-coating, and blade coating[7,8], either did not facilitate the formation of single-crystalline films, or would introduce mechanical strain during the fast crystallisation process, often resulting in a quasi-equilibrium crystalline state or even polycrystalline state[9,10].

Here, we developed a supersaturated crystallisation method for the Ph-BTBT-C_{10} single crystal growth. First, an excess amount of Ph-BTBT-C_{10} was dissolved in anisole with a high boiling point (~ 156 °C) to form a supersaturated solution. Then, the heated solution was quickly drop-cast onto the hydrophilic substrates (~ 20 °C), and the droplet would spread quickly and form a large-area air/liquid interface (Supplementary Fig. 4a). The temperature of solution was higher than the substrate, and thus a temperature gradient was formed intrinsically from the solution (high temperature) to the substrate (low temperature). Thus, the Marangoni effect caused the solution (organic molecules) to flow from the low surface tension region (high temperature) to the high surface tension region (low temperature) (Fig. 1c and Supplementary Fig. 4a), and an inward flow formed at the liquid/substrate interface (Stage I). Such a flow circulation facilitated the initial growth of the thin Ph-BTBT-C_{10} single crystals near the solution/substrate interface[11]. This process was simulated by COMSOL (Supplementary Fig. 4b). The meniscus line possessed the fastest evaporation rate (dark red point in the bottom panel in Supplementary Fig. 4b).

Marangoni flow pulled the movement of Ph-BTBT-C_{10} crystals, and the crystals at the air/liquid surface moved to the meniscus edge (red and purple dash square in images of 30 ~ 120 s in Supplementary Fig. 5); and those nanocrystals immersed near the substrate moved slowly from outside to inside (white dash rhombus in images of 30 ~ 120 s in Supplementary Fig. 5). These results were consistent with COMSOL simulations. Besides, the size of immersed crystals (white dash rhombus) increased much faster than those at the air/liquid surface (red and purple dash square). The outside-in Marangoni flow at the liquid/substrate interface facilitated the in-plane

growth of immersed thin Ph-BTBT-C_{10} crystals, while crystals at the air/liquid surface tended to be bulk. However, some crystals were too thick for Marangoni flow to pull (yellow dash square in images of 30 ~ 90 s in Supplementary Fig. 5). In addition, Marangoni effect was highly dependent on temperature gradient. A high-temperature gradient was beneficial for the deposition of large-size thin Ph-BTBT-C_{10} crystals (Supplementary Fig. 7a).

In several minutes after the drop casting, organic crystals with sizes over several hundreds of micrometres were obtained. Interestingly, clear Newton rings existed at the thin film regions and disappeared eventually (white dash circle in images of 3 ~ 60 min in Supplementary Fig. 5). We concluded that a very small amount of residual solution existed in between the Ph-BTBT-C_{10} ultrathin crystal and the substrate, forming an ultrathin liquid film underneath. Therefore, the Ph-BTBT-C_{10} crystal/solution/substrate structure was illustrated, namely a liquid bridge structure, as shown in Fig. 1c and Supplementary Fig. 4c (Stage II). Along with the slow evaporation of solution (~ 120 min), the Newton rings shrank outside-in and disappeared eventually. The thickness (t) of liquid bridge was estimated to be hundreds of nanometers using $t = (2n+1)\lambda/4$, where n is the outside-in circle number and λ is the wavelength of green light (~ 500 nm). Supplementary Figure 6a shows the mapping of liquid bridge thickness in the white square region at 5 min, 10 min, and 15 min, respectively, yielding a maximum t of ~ 800 nm at the center. During the slow evaporation process of the ultrathin liquid film underneath, the liquid bridge force at the crystal/solution interface ensured a sufficient relaxation for the strain in the Ph-BTBT-C_{10} crystals, which helped obtain large-size strain-free crystals[12,13]. Finally, large-size Ph-BTBT-C_{10} single crystals with ultra flat surfaces were grown on the SiO_2 substrates. Scanning transmission electron microscopy (STEM) characterisations were employed to assess the cross-section profile of the crystals (Supplementary Fig. 6b). A uniform Ph-BTBT-C_{10}/substrate interface was clearly seen, with a thickness of ~ 10.4 nm (corresponding to two HTH bilayers).

In conclusion, high-quality Ph-BTBT-C_{10} crystals were grown in Stage I, while the crystal relaxation occurred in Stage II. However, the evaporation rate of the solvent also influenced in the crystal growth. Bulk crystals were probable to form if the evaporation rate of the solvent was fast, while an ultraslow evaporation rate would result in superfluous crystal nuclei and the formation of polycrystals. Therefore, solvents with a high boiling point became more suitable for this method, and the substrate temperature was required to be low to ensure that the Marangoni flow can form in Stage I. The temperature of solution and substrate, solute concentration (saturated), and solvent

boiling point were adjusted to optimize this crystallisation process (Supplementary Fig. 7). The finely regulated crystallisation process occurring at the air/liquid interface with a slow evaporation rate guarantees the high-quality, strain-free Ph-BTBT-C_{10} single crystals in our experiments and the fabrication of organic transistors with an ultrahigh carrier mobility and metallic charge transport.

Supplementary Text 4. Electrical characterisations for crystalline Ph-BTBT-C_{10} transistors.

The temperature-dependent output curves of HTH Ph-BTBT-C_{10} transistors are shown in Supplementary Fig. 11a. No obvious suppression of drain current at low temperatures is observed. And the curves exhibit better linearity (Supplementary Fig. 11b), which is in agreement with the decreased contact resistance at low temperatures (Supplementary Fig. 22b).

The temperature-dependent four-probe sheet conductivities (σ_{4p}) are shown in Supplementary Fig. 12b. The value of σ_{4p} is calculated by $\sigma_{4p} = \frac{\Delta L}{Wt} \times \frac{I_D}{V_2 - V_1}$, where ΔL, W, t, V_1, and V_2 represent the distance between two voltage-sensing probes, channel width, accumulation thickness (5 nm), and four-probe voltages, respectively. The value of σ_{4p} at a sufficiently high gate voltage ($|V_G| >$ 34.5 V) exhibited a metallic temperature dependence ($\partial\sigma_{4p}/\partial T < 0$) across the entire temperature range. Supplementary Fig. 12c shows the four-probe results as a function of V_G, which stabilizes at high V_G.

Supplementary Figure 16a shows $\mu_{4p}^{\text{deri.}}$ as a function of V_G using the direct derivative method. The effective carrier mobility (μ_{eff}) was displayed in Supplementary Fig. 16b, and exhibited a typical negative temperature dependence ($\partial\mu_{\text{eff}}/\partial T < 0$)[14]. This further confirmed that our observation of the band-like temperature dependence of mobility across the entire temperature range was robust and was not dependent on the method for extracting mobility. The low reliability factor (r) is due to the shifted threshold voltage (V_T).

Supplementary Text 5. Metallic charge transport in organic field-effect transistors.

Electrical conductivity (σ) measures the ability of a material to conduct an electric current at the device level, while carrier mobility (μ) describes the speed of charge carrier (electron or hole) under the action of a unity electric field. Correspondingly, metallic charge transport is defined by a negative σ−T dependence ($\partial\sigma/\partial T < 0$), while band-like charge transport features $\partial\mu/\partial T < 0$. In

Fig. 2c in the main text, the four-point probe conductivity, σ_{4p}, in the bilayered Ph-BTBT-C_{10} exhibits a metallic temperature dependence ($\partial\sigma_{4p}/\partial T < 0$) across the entire temperature range.

In the present paper we place an emphasis on the observation of metallic charge transport (as opposed to band-like transport) for two reasons:

1) The conductivity can be measured directly, while extraction of the mobility from the FET transfer characteristics relies on assumptions, such as the validity of the ideal transistor equation. If the FET characteristics are non-ideal, as in the current devices, particularly at the lowest measurement temperatures, some uncertainty remains about the extracted mobility values. The reliability factor analysis, that was suggested in ref.[14] and used in the present paper, provides a method that reflects the ensuing uncertainty of the mobility extraction, but the extraction of conductivities is not subject to such uncertainties.

2) The notion of metallic charge transport may also be preferred from a fundamental physics point of view. It is the basis of metal-insulator transitions, which is a very important concept in condensed matter physics. Furthermore, although band-like charge transport has been observed in many high-mobility organic semiconductors, it is not always accompanied by metallic charge transport at low temperatures (Supplementary Table 2). The metallic-insulating crossover often occurs at a higher temperature than the band-hopping one. This is because it is commonly observed that the absolute value of threshold voltage ($|V_T|$) in FETs increases with decreasing temperature, especially at cryogenic temperatures[1,15–21], where some of the applied gate voltage (V_G) is needed to fill up deep trap states induced by residual disorder[17]. This V_T shift leads to a decrease of free carrier concentration ($\partial n_c/\partial T > 0$, where $n_c = (V_G - V_T)C_i/e$). Despite such a drop in the free carrier concentration the mobility extracted from the transfer characteristics above V_T may still be "band-like", in a temperature regime in which the conductivity is already decreasing with decreasing temperature. Therefore, metallic charge transport is a more stringent and difficult to reach than mere "band-like" transport. To achieve metallic charge transport to cryogenic temperatures is very challenging indeed, even in the cleanest molecular crystals[1,17,20,22–26] (Supplementary Fig. 14). Supplementary Table 2 shows the comparison of the temperature T_1, down to which band-like transport could be observed, and the temperature T_2, down to which metallic transport could be seen.

Supplementary Text 6. Delocalisation length.

Apart from the mean free path estimated from the electrical conductance and carrier concentrations, the delocalisation length can be calculated from the carrier mobility when the mobility falls within the transient localisation regime[27], especially at high temperatures (Regime I). And the power-law exponent (γ) of ~ 0.9, is in good agreement with the theoretical values for organic semiconductors by taking dynamic disorder into account[28]. Hence, the delocalisation length (d_{de}) of Ph-BTBT-C_{10} crystals is also calculated using the transient localisation scenarios, which is expressed as

$$d_{\mathrm{de}} = \sqrt{2k_{\mathrm{B}}\mu T\tau_{\mathrm{slow}}/e},$$

where the average relaxation time τ_{slow} is on the order of 100 fs[27]. The delocalisation length is dependent on the carrier mobility and temperature. The delocalisation length were ~ 4.1 nm for $\mu_{4p}^{\mathrm{Hofs.}}$ = 19 cm^2 V^{-1} s^{-1} at 300 K (V_G = −35 V), and ~ 3.6 nm for $\mu_{4p}^{\mathrm{Hofs.}}$ = 45 cm^2 V^{-1} s^{-1} at 120 K (V_G = −35 V). These results indicated that the charge carriers were delocalized on average within six Ph-BTBT-C_{10} molecules.

Supplementary Text 7. Charge carrier mobility extraction and comprehensive description on OFET device performance.

Charge carrier mobility is a key parameter in semiconductors. To extract a reliable carrier mobility and to present a comprehensive description on OFET device performance, we have adopted the following guidelines[14,29,30].

1) Four-point-probe technique should be employed to exclude the influence of contact resistance, and the device geometry should be carefully designed to avoid any underestimation or overestimation, as suggested by Prof. V. Podzorov *et al.*[30].
2) Contact resistance of the FET device should be quantitatively extracted and reported for a comprehensive description of device performance.
3) Charge carrier mobility should be extracted in the linear regime, considering that mobility obtained in the saturated regime is more sensitive to contact effect, channel length, and test temperature.
4) Charge carrier mobility should be extracted at a large $|V_G - V_T|$ regime, ensuring the formation of the accumulation layer at the semiconductor/dielectric interface and an efficient carrier injection at the electrode/semiconductor interface.
5) All information necessary for mobility evaluation should be explicitly listed, including accurate

measurements of channel geometry, the minimisation of current spreading by semiconductor patterning, and the use of gate dielectric capacitance measured at a sufficiently low frequency (f < 100 Hz).

6) When analysing charge transport properties, all μ_{4p} should be fitted at the same n_c to reliably extract the $\mu_{4p}-T$ dependence under the same carrier concentration.

7) Hall measurements are useful in supporting the observation of high FET mobilities.

Supplementary Text 8. Comparison of our Ph-BTBT-C$_{10}$ device performance with that in references.

We note that high-mobility Ph-BTBT-C$_{10}$ transistors have been reported by Hamai *et al.*[15] and Yao *et al.*[31]. Room-temperature (RT) carrier mobilities of Hamai's work and our work are summarized in Supplementary Fig. 21a. RT $\mu_{4p}^{\text{deri.}}$ and μ_{eff} in Hamai's work were 10.0 cm^2 V^{-1} s^{-1} and 5.2 cm^2 V^{-1} s^{-1}, respectively, were comparable to those in our work, 17.0 cm^2 V^{-1} s^{-1} and 5.1 cm^2 V^{-1} s^{-1}, respectively. In addition, at 80 K, $\mu_{4p}^{\text{deri.}}$ and μ_{eff} in Hamai's work were 34 cm^2 V^{-1} s^{-1} and 8.5 cm^2 V^{-1} s^{-1}, respectively, which were lower than that in our work (90 cm^2 V^{-1} s^{-1} and 42.3 cm^2 V^{-1} s^{-1}) (Supplementary Fig. 21b). The devices in Hamai's work and our work both exhibited a modest r factor, which was due to the high V_T (Supplementary Fig. 21c). We note that high mobility values have already been obtained in organic transistors, while they generally feature with large threshold voltages (Supplementary Table 3)[1,17,20,22–26].

Moreover, the metallic charge transport in Hamai's work was only maintained to 120 K (Supplementary Fig. 21d). Considering the same organic semiconductor, the measurement protocol from low to high temperatures, which has been proven to avoid disorder introduction (Supplementary Texts 10,11, and Supplementary Figs. 25,26), is a key to the observation of the metallic charge transport over a wide temperature range down to 8 K. Consequently, we also performed electrical measurements from different high temperatures (200 K, 220 K, and 240 K) to low temperature (Supplementary Fig. 28). And the lowest temperature (T_L), where the metallic transport can be maintained in these devices, increased from 70 K to 100 K. In Hamai's work, the electrical temperature-dependent characteristics were performed from 300 K to low temperature (confirmed by Hamai in private communication). The yellow dot in Supplementary Fig. 21e represents that more disorder was introduced into their Ph-BTBT-C$_{10}$ devices at 300 K.

In addition, r factor in Yao' work was estimated to ~ 92%. We also fabricated devices with transferred electrodes. A high r factor (~ 90%) and 2p μ_{eff} of 14 $cm^2\ V^{-1}\ s^{-1}$ were obtained at room temperature, which were comparable with that in Yao's work. However, devices with transferred electrodes were found to be insufficiently robust for the low-temperature measurements, compared with those with evaporated electrodes (Supplementary Text 9).

Supplementary Text 9. Devices using transferred electrodes and PVC-modified interfaces.

We employed evaporated source drain electrodes as described in Methods section, and also used transferred Au electrodes. Devices with laminated contacts[31] have been reported to achieve cleaner electrode/semiconductor interfaces, small V_T and low contact resistance at room temperature. We found that this was indeed the case for our Ph-BTBT-C_{10}; however, the contact resistance (R_c) of devices with laminated contacts degraded obviously with the decreasing temperature, making them unsuitable for our temperature-dependent charge transport study (Supplementary Figs. 22a,b). R_c change in evaporated devices was much smaller within 8 ~ 240 K. Thus, this temperature range was chosen to investigate the charge transport in Ph-BTBT-C_{10} transistors with evaporated electrodes. We potentially attribute this to a reduced robustness of the laminated contacts to thermally-induced mechanical stress induced by mismatch of the thermal expansion coefficients of the different layers of the devices.

To investigate the reason for the different R_c−T dependence in samples with evaporated and transferred electrodes, cross-sectional STEM characterisations were performed to assess the Au/Ph-BTBT-C_{10} interfaces for both samples with evaporated and transferred electrodes (Supplementary Fig. 22c). For the evaporated sample, the evaporation rate of Au electrodes was controlled at an ultraslow speed of ~ 0.03 Å s^{-1} in the initial ~ 5 nm, which enabled the Au diffusion length within one-bilayer-thick region. This evaporation process for the top source-drain contacts not only ensures the intact charge transport layers, but also enables yielded robust electrodes with low contact resistance[32] down to low temperatures. For the laminated electrodes such an inter-diffused layer is absent, i.e. in the STEM image the Ph-BTBT-C_{10} film appears to have its original, ~ 10 nm thickness, corresponding to two molecular bilayers. This is likely to lead to poorer film adhesion under the low temperature measurement cycles.

Low-κ polyvinyl cinnamate (PVC) was also utilized to modify the semiconductor/dielectric interface[33]. The obtained transfer curves measured for 4 times exhibited negligible hysteresis

(Supplementary Fig. 23a). However, $\mu_{4p}^{Hofs.}$ of PVC device was lower than that using SiO_2 (Supplementary Figs. 23c,d). AFM measurements were carried out to assess the roughness of the dielectric interface (Supplementary Figs. 23e,f). The roughness of SiO_2 was only ~ 0.99 Å, while that of PVC increased up to ~ 3.97 Å. This increased roughness resulted in a low carrier mobility.

When combining laminated Au electrodes and interface engineering, the device exhibited negligible hysteresis for 30 consecutive scans in the air at room temperature (Supplementary Fig. 23b). We attributed the small V_T and negligible V_T shift to the high-quality semiconductor/dielectric interface modified by PVC and the clean electrode/semiconductor interface obtained by transferred Au, both leading to a low density of shadow trap states at these two key interfaces in transistors. However, as aforementioned, the contact resistance for devices with laminated Au increased markedly at low temperatures, and the roughness of PVC was larger than SiO_2. Thus, in this work, evaporated electrodes and ultra flat SiO_2 were preferred to achieve metallic transport over a wide temperature range with a high carrier mobility in our Ph-BTBT-C_{10} transistors.

Supplementary Text 10. Photoluminescence spectroscopy.

Photoluminescence spectroscopy (PL) is a sensitive probe to study the disorder-induced dynamic charge transfer excitonic states in organic semiconductors[34]. Disorder was introduced into the Ph-BTBT-C_{10} film through electrical treatments at room temperature. Temperature-dependent PL spectra were measured for the same Ph-BTBT-C_{10} sample before and after the disorder introduction (Supplementary Fig. 25a). Typical excitonic peaks within 2.8 ~ 3.2 eV were observed at all temperatures. In addition, the *0−0* transition is supposed to be strictly forbidden in H-aggregated single crystals without disorder[34]. Thus, the intensified *0−0* peak confirmed the disorder introduction. Furthermore, time-resolved PL (TRPL) measurements were performed on the Ph-BTBT-C_{10} samples at the *0−1* peak (393 nm) (Supplementary Fig. 25b). The TRPL curves could be well-fitted by a biexponential function, from which the decay time of τ_1 and τ_2 were extracted (Supplementary Fig. 25c). The value of τ_2 was larger in the disordered sample than the pristine one, which indicates more trap states inducing repeated trapping and detrapping processes to prolong the exciton lifetime. These results confirmed that extra disorder was indeed introduced into the HTH Ph-BTBT-C_{10} films by the co-regulation of temperature and electric field, which was in good agreement with the conclusion in Supplementary Fig. 26.

In addition, the disorder introduction was irreversible on the timescale of our electrical measurements, proven by the large threshold voltage in low-temperature transfer curves once the disorder introduced at high temperatures.

Supplementary Text 11. Introduction of disorder in Ph-BTBT-C_{10} single crystals.

Metal-insulator transition (MIT) is one of the most fundamental problems in condensed-matter solids. Generally, an MIT is described by two well-known concepts: i) Mott transition occurs when the Coulomb repulsion between carriers dominates over the kinetic energy; ii) Anderson localisation matters when the increasing degree of disorder turns the delocalized electronic states at the Fermi energy into localized states in disordered systems[35,36]. Until now, rare organic semiconductors are known where the MIT can be solely attributed to disorder-induced localisation[35].

The density of deep trap states ($N_{\mathrm{trap}}^{\mathrm{de}}$) can be tuned finely by the electrical operation, which can be calculated by $N_{\mathrm{trap}}^{\mathrm{de}} = V_{\mathrm{T}}C_{\mathrm{i}}/e$. Due to the V_{T} shift at high temperatures, we performed cyclical electrical measurements from 100 K to 300 K, and then back to 100 K (Supplementary Fig. 26a). During the warming-up process, the value of V_{T} first remained nearly temperature-independent from 100 K to 200 K, and then increased at higher temperatures (from 200 K to 300 K). However, V_{T} maintained nearly unchanged in the whole cooling process, indicating that the measurement of the device, i.e. the application of source-drain and electric fields at high temperature, introduces the existence of a certain density of deep trap states that remains even when re-cooling the device. Thus, the disorder was successfully introduced by the co-regulation of temperature and electric field, which was further characterized by PL spectroscopy (Supplementary Text 10).

We do not currently fully understand the nature of the deep trap states that are introduced when measuring the device at temperatures above 200 K and cause the irreversible increase in V_T shown in Supplementary Fig. 26a. We would like to propose the following tentative explanation that is consistent with our experimental observations:

In devices with laminated contacts (Supplementary Text 9), we do not observe such an increase in V_{T} when measuring at or near room temperature. It is only observed in the devices with evaporated contacts. This suggests a potential defect generation mechanism that is associated with the interface between the organic semiconductor and the top source-drain electrodes. In devices with laminated contacts, this interface is likely to be abrupt with little metal interdiffusion into the

organic semiconductor layer. At such an abrupt interface, metal ions are less likely to diffuse into the organic semiconductor channel when applying voltages to the device. In contrast, in the devices with evaporated contacts, there is some metal interdiffusion into the semiconductor, which is clearly evident from Supplementary Fig. 22c. When applying voltages to the device near room temperature, metal ions might further diffuse into the organic semiconductor and generate trap states in the semiconductor channel. When applying voltages to the device only at low temperatures, however, such metal diffusion into the organic semiconductor layer might be suppressed by the temperature activated nature of the diffusion process.

Although the devices with laminated contacts can be measured stably with low V_T at room temperature, we cannot use them for our experiments, as laminated contacts are insufficiently robust for the low-temperature measurements, potentially due to the thermal stress experienced during cooling and insufficient adhesion of the metal contacts. On the other hand, our devices with evaporated contacts provide sufficient robustness for low-temperature measurements, but exhibit a V_T increase when measuring them at temperatures above 200 K. The approach adapted in our work of using evaporated contacts but avoiding measurements near room temperature is pragmatic and has allowed us to observe the excellent charge transport properties at low temperatures. In the future, more extensive contact optimisation might be able to discover metallisation techniques that combine sufficiently strong mechanical adhesion to allow low-temperature measurements and a high interface quality to suppress voltage-induced metal interdiffusion at high temperatures in these molecular bilayers with vdW bridges.

Supplementary Text 12. Observation of disorder-driven MIT.

The Anderson and Mott transitions are actually described within one conceptional framework of energy arguments, illustrated in Supplementary Fig. 27a. When the Ioffe–Regel criterion, $k_F \bullet l_e \sim 1$, where k_F and l_e is the Fermi wave vector and mean free path, respectively, is satisfied, an MIT can be observed. For Mott transition, a high n_c is required to generate a strong electron-electron/hole-hole Coulomb correlation. This Coulomb correlation can be represented by the Wigner-Seitz radius (r_s)[37]. When $r_s >> 1$, the Coulomb correlation dominates the kinetic energy, and a Wigner crystal forms[38,39]. When the Coulomb correlation is negligible, the introduction of disorder can also turn the delocalized carriers to be localized, and result in a transiti metallisation

on from metallic to insulating. In general, it is difficult to observe the continuous transition process between metallic and insulating states that is driven by the disorder.

The aforementioned method of disorder introduction in Ph-BTBT-C_{10} offered the possibility for the observation of continuous disorder-driven MIT. Supplementary Figure 27b shows the conductance as a function of temperature for the sample with disorder introduced at 200 K (the raw transfer curves are shown in Supplementary Fig. 28a). At low V_G (−10 ~ −17 V), a complete insulating behaviour was observed while metallic transport occurred at higher V_G (−17 ~ −25 V). The critical V_G for this transition was ~ −17 V. We further showed the field-temperature phase diagram for $|\lg(R/R_T)|$ in Supplementary Fig. 27c, revealing a "fan-shape" structure that was widely observed for MIT[40].

To confirm that the observed MIT was driven by disorder, we further studied the conductance of Ph-BTBT-C_{10} with various disorder levels. The disorder level was tuned *via* carrying out electrical operations at different initial temperatures (T_{in}). The cycle measurements were performed as follows: i) from 200 K to 60 K (T_{in} = 200 K); ii) from 220 K to 60 K (T_{in} = 220 K); and iii) from 240 K to 60 K (T_{in} = 240 K). In Supplementary Fig. 28b, typical MIT can be observed in all three cycle measurements. For instance, in the device measured using the initial temperature of 220 K, an insulating behaviour was observed when V_G was in the range of −10 ~ −18 V. Inversely, at higher V_G (−18 ~ −25 V) the conductance exhibited a transition to a metallic behaviour. With the increasing disorder level (T_{in} increases from 200 K to 240 K), the critical V_G changed from −17 V to −19 V (corresponding to the increased V_T). Hence, we developed one feasible approach to introduce disorder by co-regulating the temperature and electric field.

Moreover, the conductance also displayed a decreased trend with higher disorder densities. The critical conductance in units of e^2/h (h and e is the Planck's constant and the elementary charge, respectively) was ~ 0.26, 0.11, and 0.07 for the measurements using the initial temperatures of 200 K, 220 K, and 240 K, respectively. In literature, the critical conductance in low-disordered 2D semiconducting systems was in the range from 1/3 to 2 in units of e^2/h[41–43]. Our results displayed a derivation, which was due to the introduction of disorder. And this derivation became more profound when increasing disorder densities[42,44]. Thus, we conclude that disorder in organic semiconductors can restrain carrier delocalisation and lead to a shorter localisation length.

The key of a temperature-immune OFET is the zero-temperature coefficient (ZTC) point, where the drain current and electrical conductivity remain nearly unchanged at various

temperatures. In our demonstration, after the disorder introduction in the pristine Ph-BTBT-C_{10} bilayer crystal that initially possessed wide-temperature-range metallic charge transport, we can clearly observe an MIT, featuring a crossover point in the temperature-dependent transfer curves. Hence, we can directly realize a temperature-immune OFET at the gate voltage where that crossover point locates (Supplementary Fig. 29).

Supplementary Text 13. Threshold voltage extraction in OFETs.

To assess V_T in OFETs with strong μ−V_G dependence, the second derivative method was employed. The value of V_T is determined where the maximum of the derivative of the transconductance (i.e., $\frac{\mathrm{d}g_\mathrm{m}}{\mathrm{d}V_\mathrm{G}}$) first appears[45,46]. We took the transfer curve at 140 K as an example (Supplementary Fig. 30a). Supplementary Figs. 30b,c represent the first derivative ($g_\mathrm{m} = \frac{\mathrm{d}I_\mathrm{D}}{\mathrm{d}V_\mathrm{G}}$) and second derivative ($\frac{\mathrm{d}g_\mathrm{m}}{\mathrm{d}V_\mathrm{G}}$) of the transfer curve, respectively. And the first maximum of $\frac{\mathrm{d}g_\mathrm{m}}{\mathrm{d}V_\mathrm{G}}$ was obtained at $V_G = V_T = -15.4$ V. Supplementary Figure 30d shows the temperature dependence of threshold voltage extracted by the second derivative method.

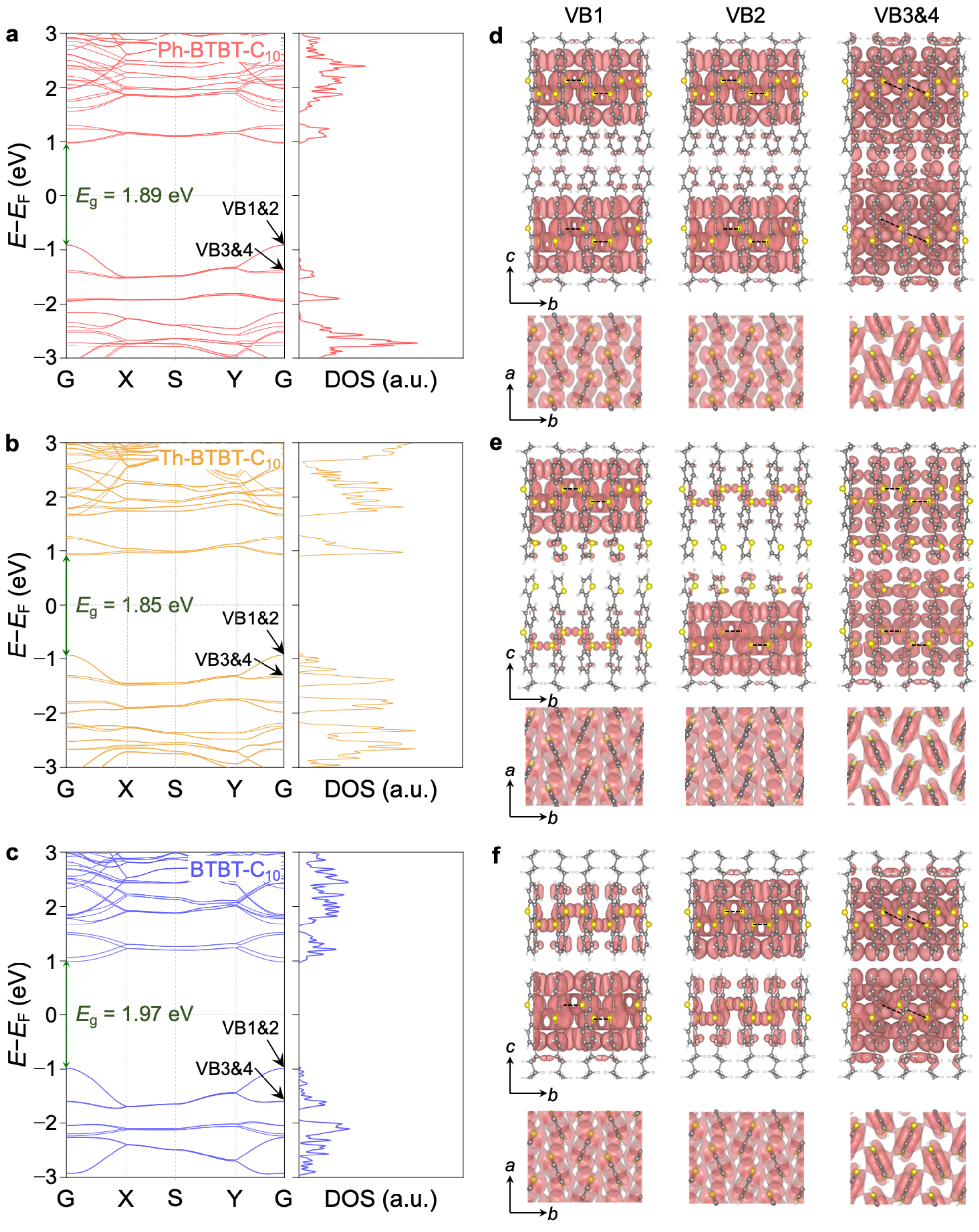

Supplementary Fig. 1 | Band structures and wavefunction norms of Ph-BTBT-C$_{10}$, Th-BTBT-C$_{10}$, and BTBT-C$_{10}$. a-c, The band structures and density of states of Ph-BTBT-C$_{10}$, Th-BTBT-C$_{10}$, and BTBT-C$_{10}$ bilayers. **d-f**, Visualized wavefunction norms of valence band maxima (VB1−4) labelled in (**a-c**) with an isosurface level of 0.0002 e/Bohr^3, respectively. The black dashed lines in (**d-f**) are corresponding to conducting channels.

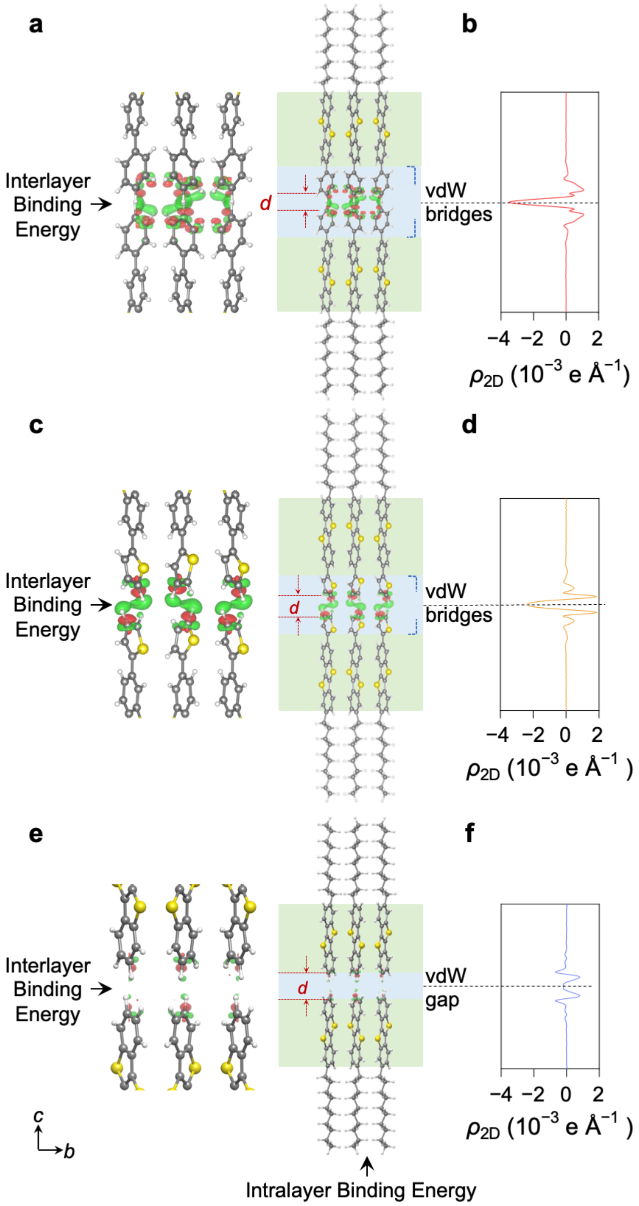

Supplementary Fig. 2 | Differential charge densities (DCD) of Ph-BTBT-C10, Th-BTBT-C10, and BTBT-C10. a,**c**,**e**, DCD of head-to-head (HTH) Ph-BTBT-C10, Th-BTBT-C10, and BTBT-C10 bilayers, with an isosurface level of 0.0003 e $Bohr^{-3}$. The blue region (van der Waals bridges regions) of the right panel is zoomed in and shown in the left panel. The green region represents the BTBT molecular cores. **b**, Line profile of DCD along the *c* direction for (**a**). The range of the ordinate is corresponding to the colored region in (**a**). **c**,**d** and **e**,**f** show the DCDs and line profiles of Th-BTBT-C10 and BTBT-C10, respectively.

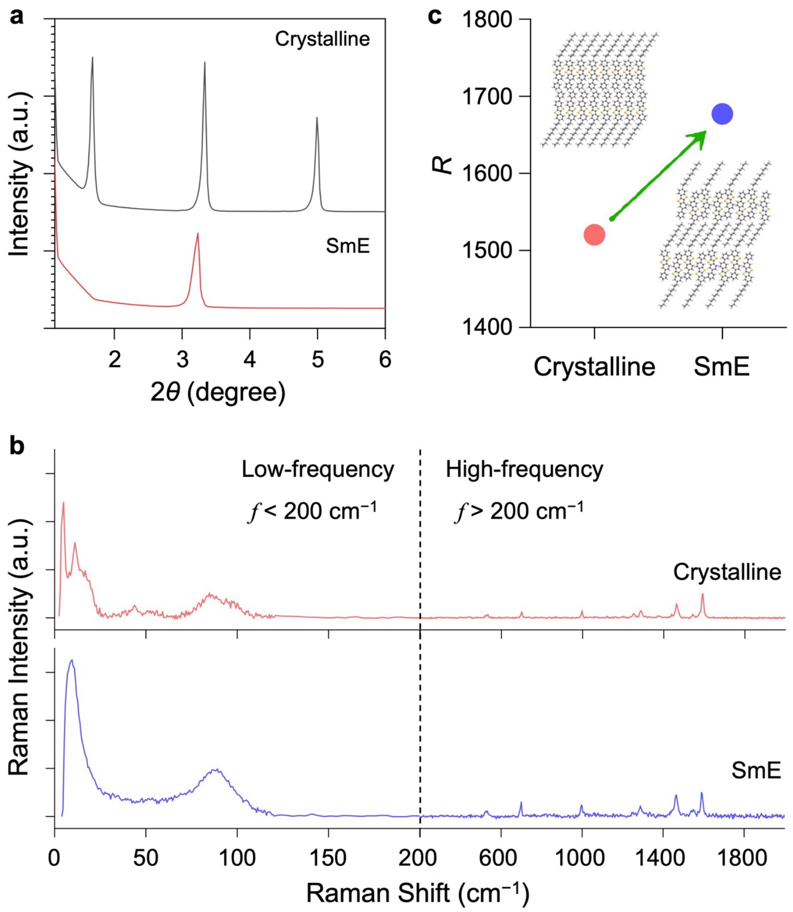

Supplementary Fig. 3 | Raman spectra of crystalline and smectic E (SmE) Ph-BTBT-C10 powder samples. a, X-ray diffraction (XRD) patterns for the crystalline and SmE Ph-BTBT-C10 powder samples. **b**, Raman spectra for crystalline and SmE Ph-BTBT-C10 powder samples. **c**, The value of *R* for crystalline and SmE Ph-BTBT-C10. The strength of dynamic disorder (*ΔJ*/*J*) in molecular semiconductors can be estimated from the spectroscopically available quantity *R* by $R = \int_{\mathrm{LF}} I \mathrm{d}\omega / \int_{\mathrm{HF}} I/\omega \mathrm{d}\omega$ and $(\frac{\Delta J}{J})_{\mathrm{Raman}} \propto \sqrt{R}$, where *R* for crystalline Ph-BTBT-C10 (1520) is lower than that for the SmE one (1677).

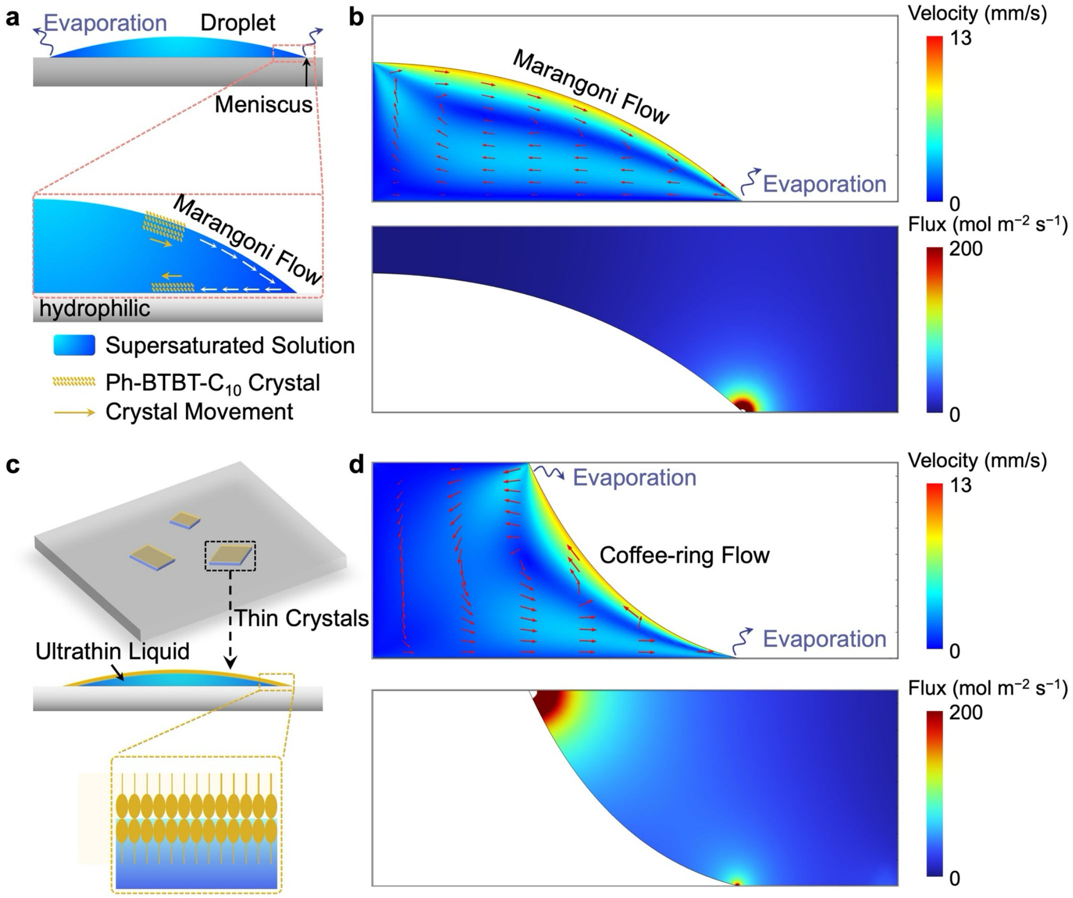

Supplementary Fig. 4 | Illustration of the supersaturated crystallisation method and corresponding COMSOL simulations. a, Illustration of the droplet on the hydrophilic substrate (Stage I). Marangoni flow is labelled, pulling the movement of Ph-BTBT-C_{10} molecules and crystals. **b**, COMSOL simulation results of velocity distribution (upper) and evaporation flux (bottom). **c**, Formation of the liquid bridge in between Ph-BTBT-C_{10} crystals and substrate (Stage II). Ultraflat Ph-BTBT-C_{10} crystals were finally deposited on the substrate. **d**, COMSOL simulation results of velocity distribution (upper) and evaporation flux (bottom).

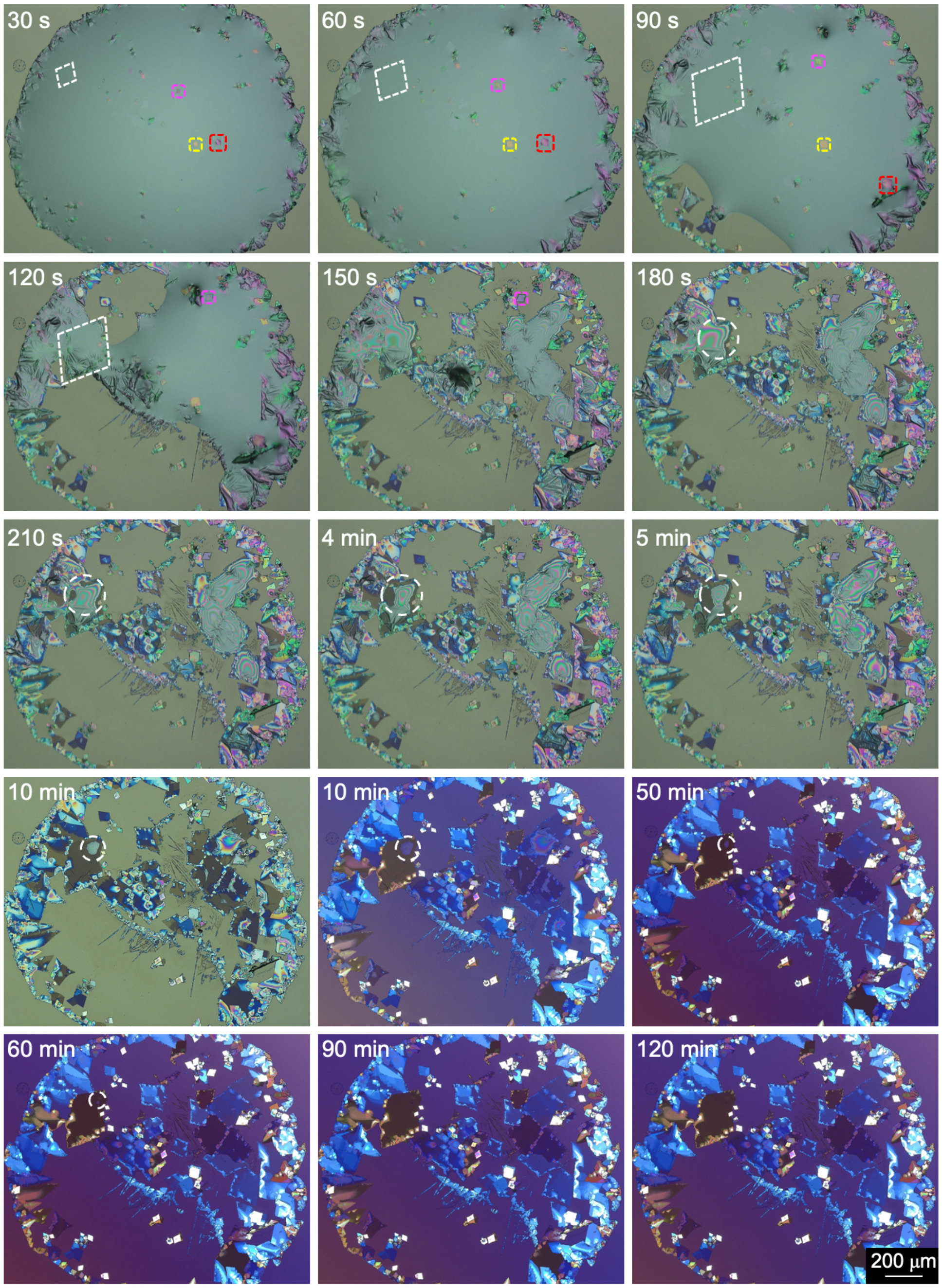

Supplementary Fig. 5 | Real-time Ph-BTBT-C$_{10}$ crystal growth using the supersaturated crystallisation method. Optical (3 s ~ 10 min) and polarized optical (10 ~ 120 min) images were taken during this solution process. The evaporation of solution on substrate finished within ~ 3 min, while the Newton rings shrank outside-in slowly due to the slow evaporation of solution underneath Ph-BTBT-C$_{10}$ crystals (3 ~ 120 min).

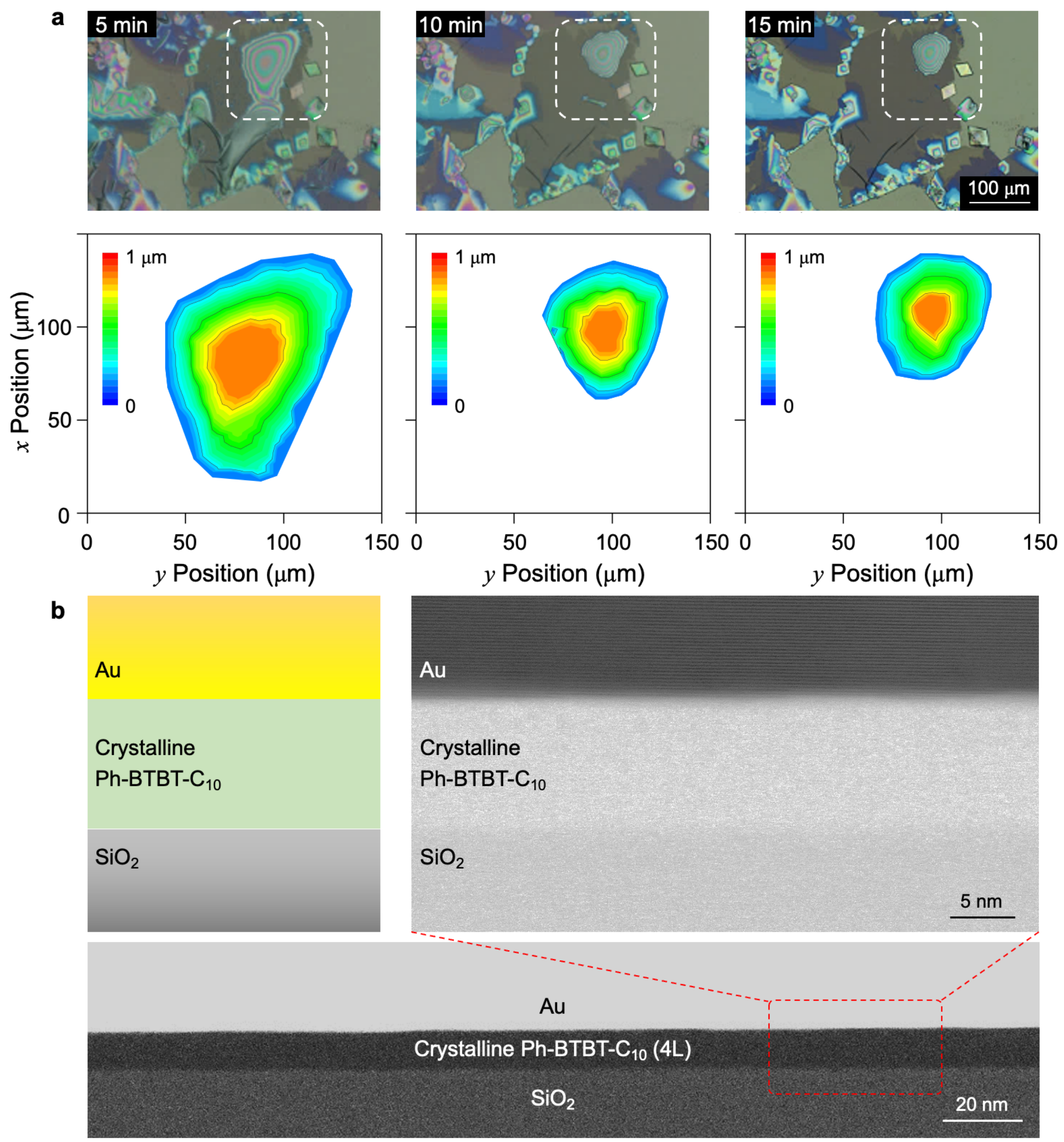

Supplementary Fig. 6 | Real-time mapping of liquid bridge thickness and scanning transmission electron microscopy (STEM) images of Ph-BTBT-C$_{10}$ crystal. a, Optical images and corresponding thickness mapping of liquid bridge in the white square region at 5 min, 10 min, and 15 min, respectively. **b**, Schematic diagram of the sample structure for STEM measurement (top left), cross-section high angle annular dark-field STEM image (bottom), and zoomed bright field STEM image in the red square region (top right). Au electrode was transferred to the crystal to keep the crystal intact.

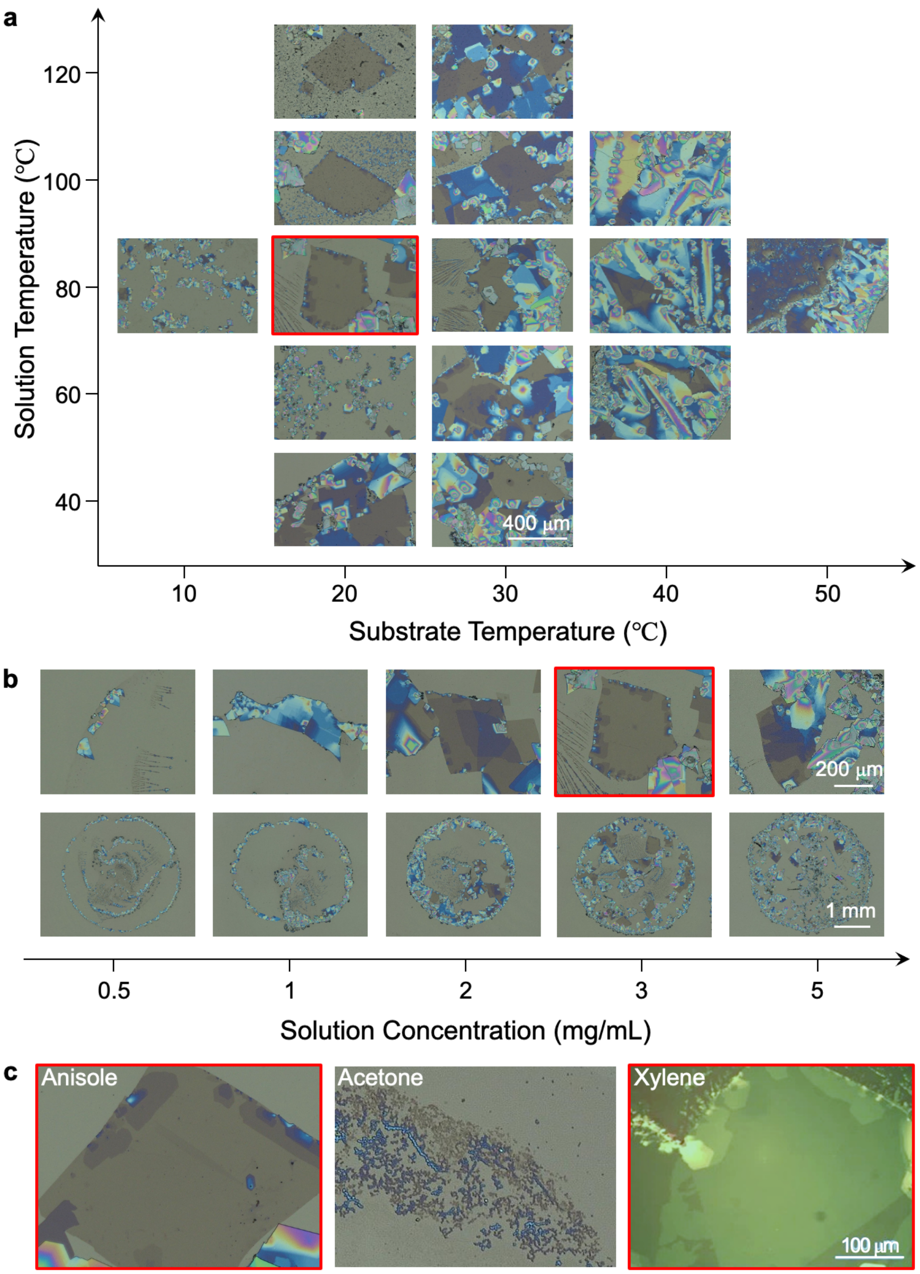

Supplementary Fig. 7 | Optimisations for Ph-BTBT-C_{10} crystal growth. a, Influence of temperatures of solution and substrate on the Ph-BTBT-C_{10} crystal growth. **b**, Influence of solution concentration on the Ph-BTBT-C_{10} crystal growth. **c**, Influence of boiling point on the Ph-BTBT-C_{10} crystal growth.

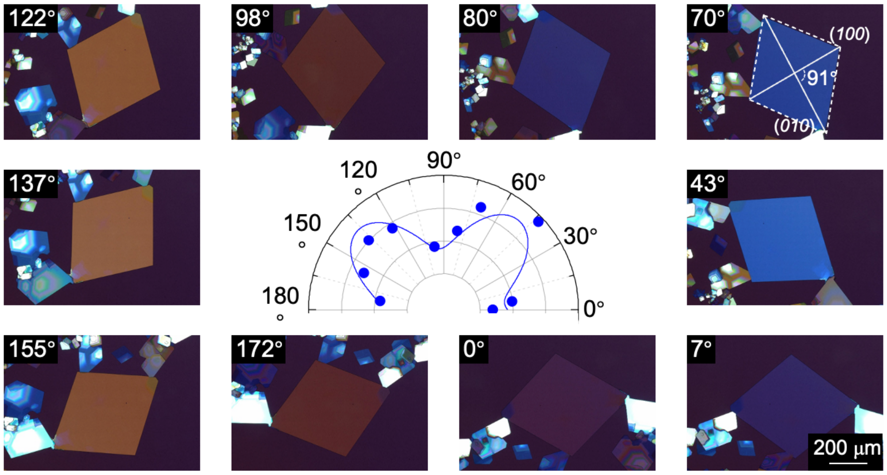

Supplementary Fig. 8 | Cross-polarized optical microscopy images at various polarization angles. The brightness of the crystals changes along with the sample rotation, which shows a good symmetry as a function of the polarization angles.

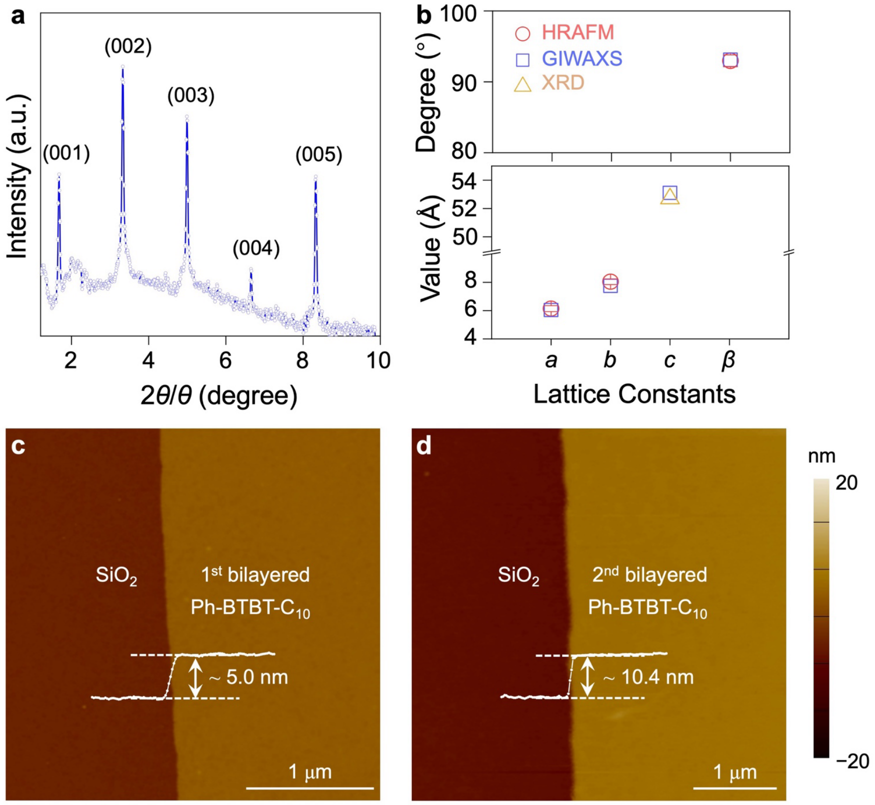

Supplementary Fig. 9 | Summary of the lattice parameters and thicknesses of Ph-BTBT-C_{10} films. **a**, XRD spectra for the crystalline Ph-BTBT-C_{10} films. Typical (*00l*) peaks are shown and the corresponding *d*-spacing is calculated to be ~ 5.4 nm, which is close to the theoretical value and atomic force miscroscopy (AFM) data. **b**, Summary of the experimental lattice parameters (*a*, *b*, *c*, and *β*) of Ph-BTBT-C_{10} obtained from high-resolution AFM (HRAFM), grazing incidence wide-angle X-ray scattering (GIWAXS), and XRD. Error bars in HRAFM data represent the standard deviation of lattice parameters, calculated from measurements at 10 different positions. **c**,**d**, AFM images of the first and second bilayer Ph-BTBT-C_{10} films, respectively.

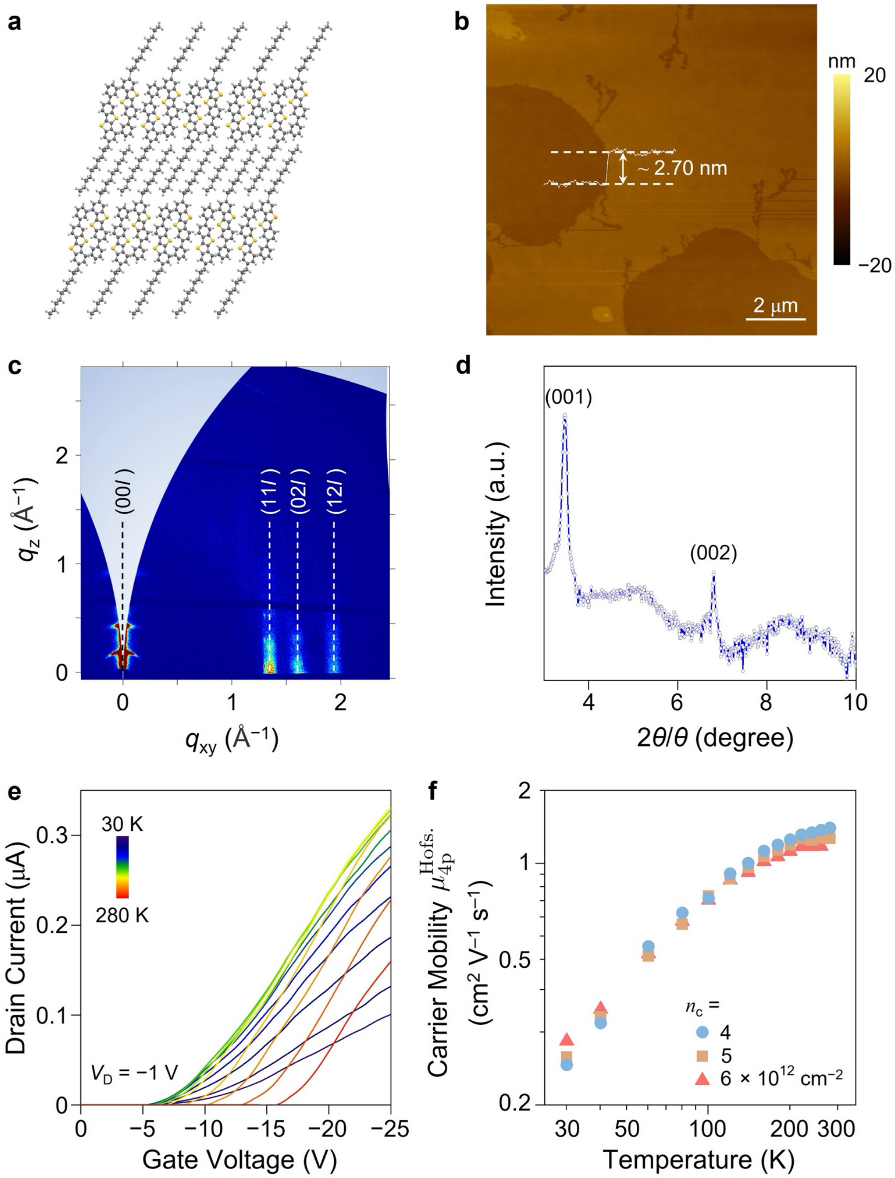

Supplementary Fig. 10 | Film property and electrical characterisations for SmE Ph-BTBT-C_{10} films. a, Illustration of head-to-tail (HTT) interpenetrated molecular packing in SmE Ph-BTBT-C_{10}. **b**, AFM image. The SmE sample showed a height of ~ 2.7 nm for the observed step, corresponding to the length of one Ph-BTBT-C_{10} molecule. **c**, GIWAXS image. Fewer spots can be observed, showing a relatively lower ordered molecular packing, compared with crystalline Ph-BTBT-C_{10} films. **d**, XRD spectra. The *d*-spacing corresponding to the observed peaks is ~ 2.65 nm, in good agreement with the AFM data. **e**, Temperature-dependent transfer curves in the linear regime. **f**, Carrier mobility as a function of temperature and carrier concentration.

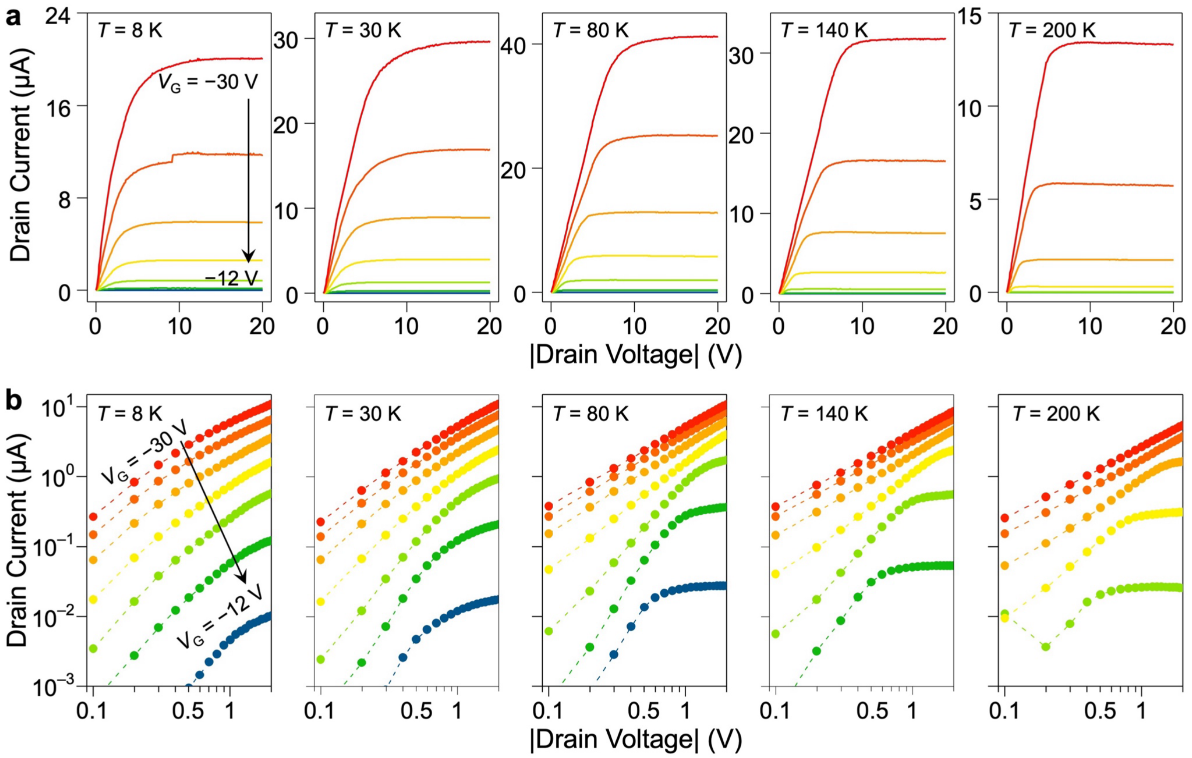

Supplementary Fig. 11 | Output curves for HTH Ph-BTBT-C_{10} OFET. The output characteristics in linear (**a**) and logarithmic (**b**) scale. No obvious suppression is observed in the drain current at the low drain voltages, especially in low temperatures.

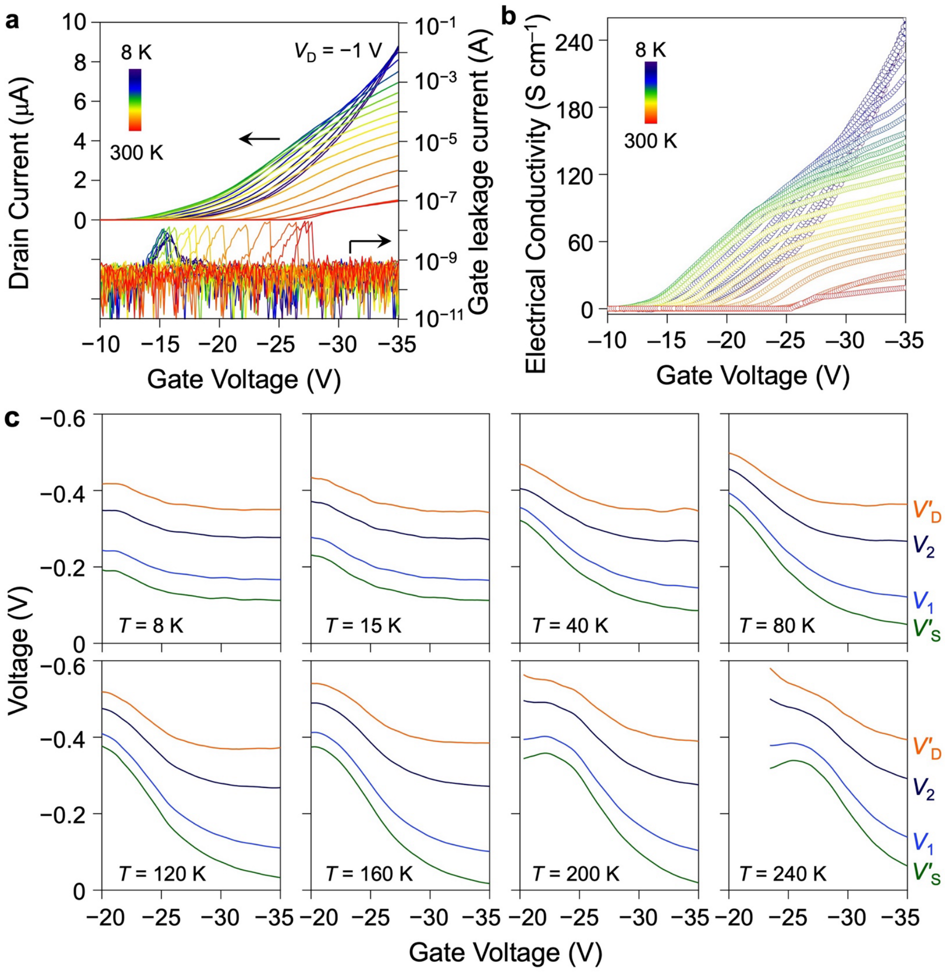

Supplementary Fig. 12 | Electrical characterisations for the Ph-BTBT-C_{10} OFET. a, Linear transfer curves and leakage currents at a drain voltage V_D of −1 V. **b**, The values of four-point-probe electrical conductivity (σ_{4p}) plotted as a function of gate voltages (V_G). **c**, V_G dependence of the potentials at sense probes V_1, V_2, and inside of the source V_S' and the drain V_D', where V_S' and V_D' are estimated by the linear extrapolation from the sense probes.

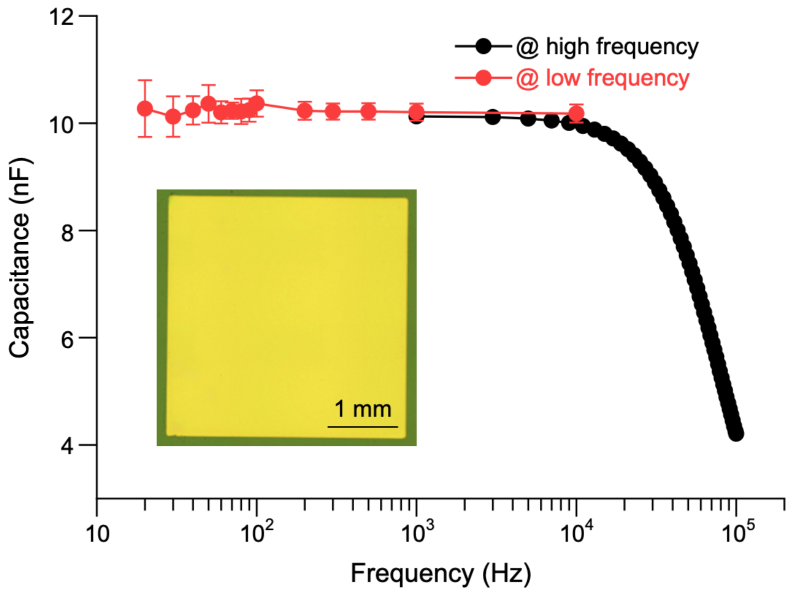

Supplementary Fig. 13 | Unit capacitance (C_i)-frequency (f) dependence for SiO_2 substrates used in our devices. Error bars represent the standard deviation from 20 data points measured for the same sample, with the center representing the mean value. Inset shows a plane-parallel capacitor with a size of 4.0 × 4.0 mm^2 to measure C_i experimentally. At low frequencies (f = 100 Hz), the capacitance was 10.2 ± 0.2 nF. A C_i of 63.75 nF cm^{-2} was used in the mobility calculation.

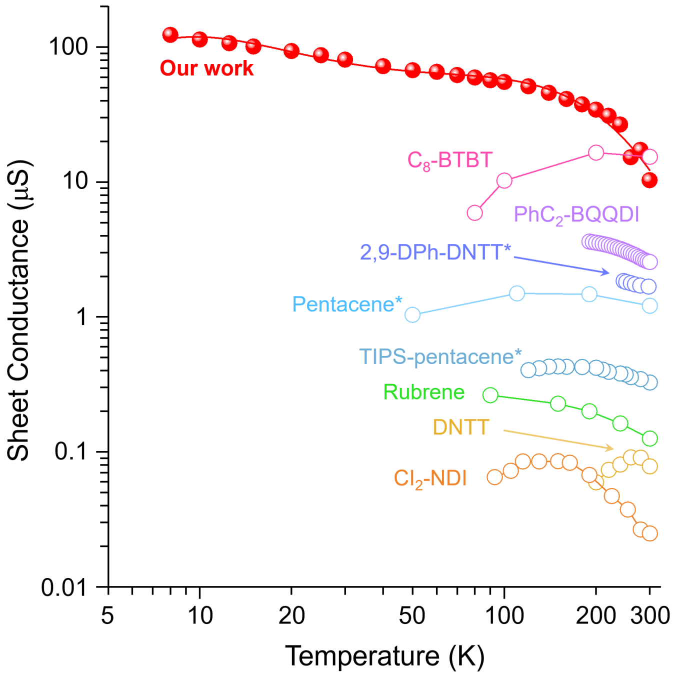

Supplementary Fig. 14 | Summary of the electrical conductance-temperature dependence in high-mobility organic semiconductors. The sheet conductance G labelled by * was extracted by the two-probe technique, the others were obtained by the four-point-probe technique[1,16,17,20,22,24,26,49]. The measurement parameters for these materials are shown in the Supplementary Table 2. Note that electrical conductance ($G = (L/W)\cdot(I_D/V)$) is in direct proportion to conductivity ($\sigma = (L/Wd)\cdot(I_D/V)$), where L, W, d, and V represent the distance between electrodes, channel width, accumulation thickness, and voltage, respectively. Hence, electrical conductance is used for comparison here instead of conductivity (σ) since the thicknesses of accumulation layer are not given in some references.

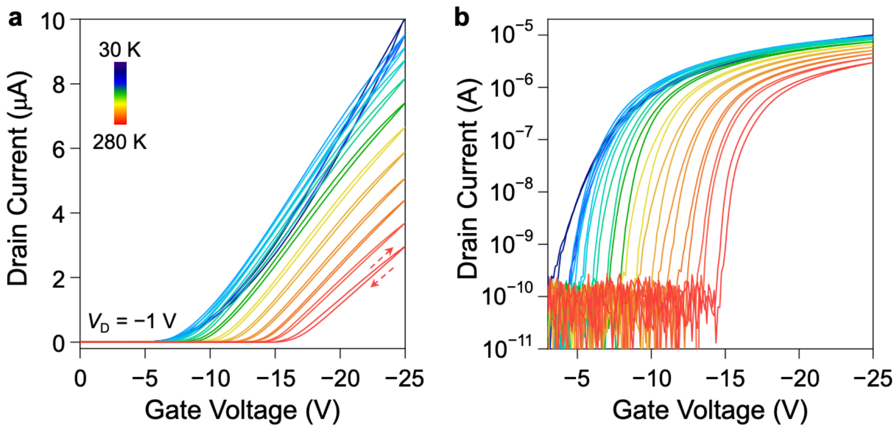

Supplementary Fig. 15 | Hysteresis in a HTH Ph-BTBT-C_{10} transistor. Linear transfer curves in linear (**a**) and logarithmic (**b**) coordinates of a Ph-BTBT-C_{10} device. The sample was measured within a temperature range from 30 K to 280 K. The anticlockwise hysteresis was very slight at various temperatures, and no apparent hysteresis-temperature dependence was observed.

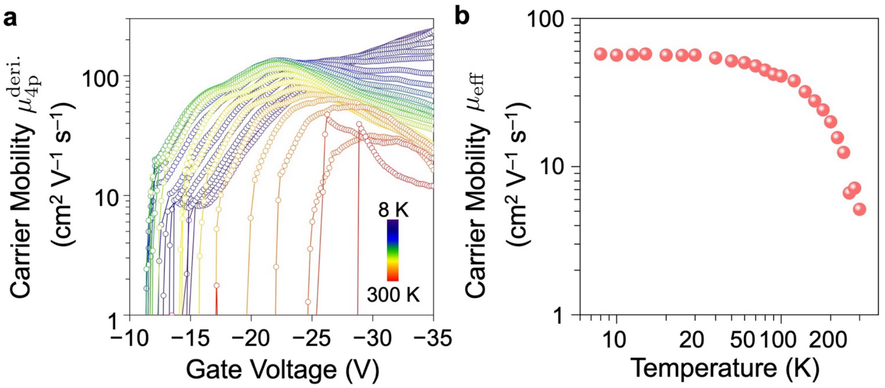

Supplementary Fig. 16 | Carrier mobility extraction using direct derivative method. a, Temperature-dependent carrier mobility extracted using direct derivative method, $\mu_{\mathrm{eff}}^{\mathrm{deri.}}$, plotted as a function of gate voltage. **b**, Effect carrier mobility, μ_{eff}, ploted as a function of temperature. The μ_{eff} at 80 K and room temperature are 44.7 and 5.1 cm^2 V^{-1} s^{-1}, respectively.

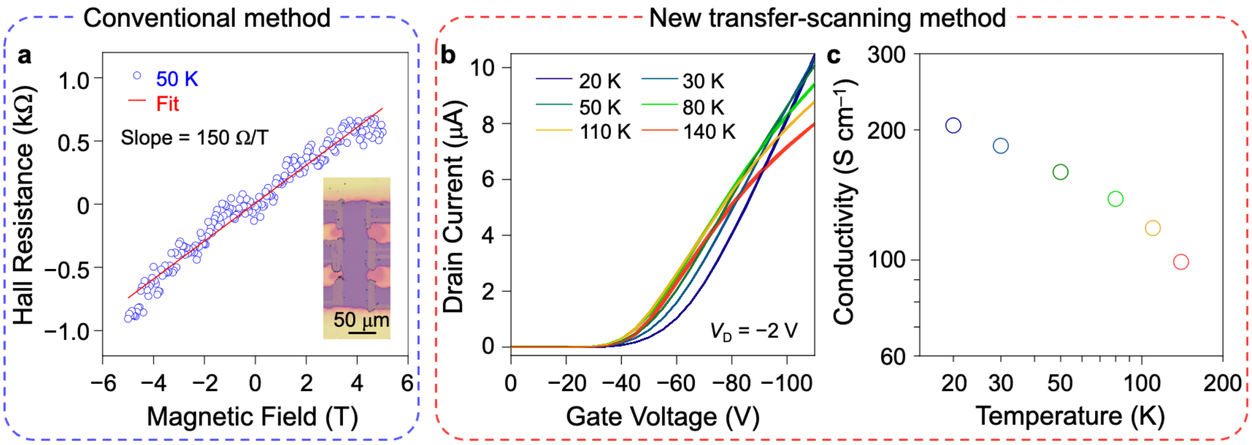

Supplementary Fig. 17 | Temperature-dependent Hall measurements employing conventional magnetic-field-sweeping method and transfer-scanning method for the Ph-BTBT-C$_{10}$ FET. a, Hall resistance (R_{xy}) of Ph-BTBT-C$_{10}$ at 50 K (points) and linear fitting (red line), which was obtained by conventional Hall testing method with constant current *I*. Inset shows the optical image of the Hall device. The Hall carrier concentration $n_{Hall} = (eR\ H)^{-1}$, derived from the Hall coefficient $R_H = V_H/IB$, the longitudinal conductance calculated by the four-point-probe voltage drop recorded simultaneously, allowing to extract the Hall mobility μ_{Hall} ~ 85 cm^2 V^{-1} s^{-1} ($n_{Hall} = 4.2 \times 10^{12}$ cm^{-2}). The dielectric is 250 nm-thick SiO_2. **b**, Transfer curves at magnetic fields (*B*) changing from −6 T to 6 T with a step of 0.3 T at various temperatures. No obvious change in the transfer curves was observed. It indicates nearly identical current through our sample during the entire Hall measurements, which is equivalent to the conventional Hall measurements by applying constant drain current at a fixed gate voltage. The drain current (I_D) and the Hall voltage (V_H) were detected simultaneously to obtain the Hall resistance ($R_{xy} = V_H/I_DB$). The dielectric is 200 nm-thick SiO_2. **c**, The longitudinal electrical conductivity plotted as a function of temperature, which was calculated by the four-probe voltage at $V_G = -110$ V. A Hall mobility at 50 K of ~ 78 cm^2 V^{-1} s^{-1} (Fig. 3b in the main text) well matches that obtained from conventional Hall measurements. Therefore, these results validate the reliability of our Hall testing method.

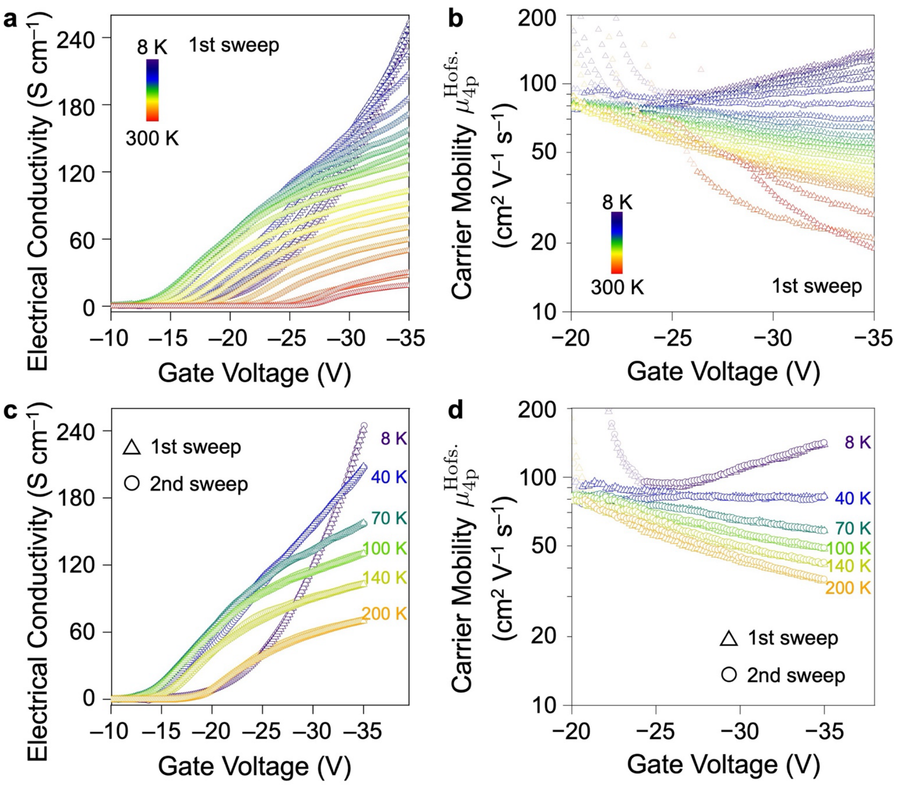

Supplementary Fig. 18 | Repeated gate sweeps of the Ph-BTBT-C_{10} sample in Fig. 2 in main text. a,**b**, V_G dependence of electrical conductivity (**a**) and carrier mobility (**b**) in the first sweep of the Ph-BTBT-C_{10} sample. The charge transport analysis in the main text is based on the results of the second sweep, as shown in Fig. 3a and Supplementary Fig. 12b. **c**,**d**, Twice V_G sweeps of this Ph-BTBT-C_{10} sample, which displays a good reproducibility.

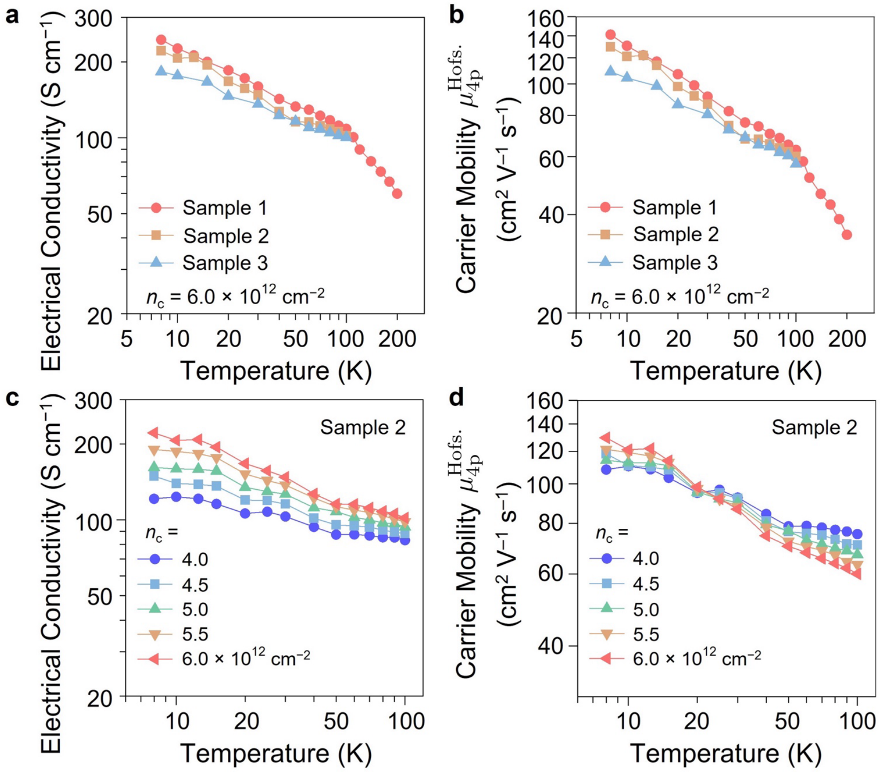

Supplementary Fig. 19 | Electrical characterisations for three Ph-BTBT-C_{10} samples. a,**b**, The temperature dependence of electrical conductivity (**a**) and carrier mobility (**b**) in three samples, all exhibiting metallic charge transport down to 20 K with ultrahigh electrical conductivity > 100 S cm^{-1} and ultrahigh carrier mobility > 100 cm^2 V^{-1} s^{-1}. Sample 1 is the best and described in main text (Figs. 2,3). **c**,**d**, Temperature-dependent electrical conductivity (**c**) and carrier mobility (**d**) at various carrier concentrations, n_c, in sample 2.

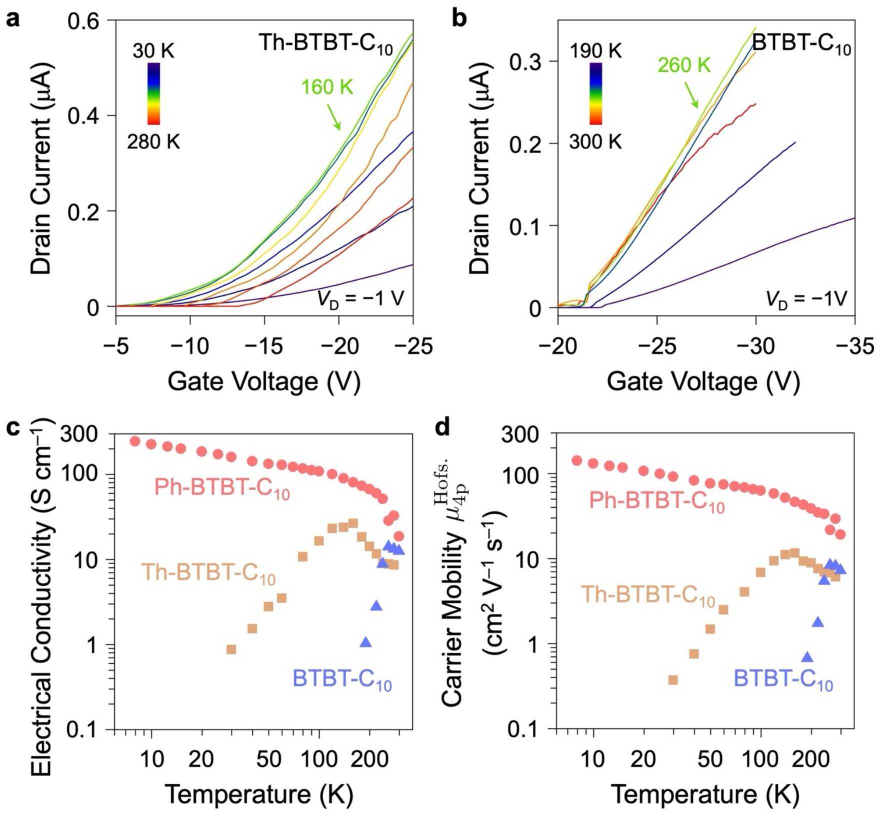

Supplementary Fig. 20 | Electrical characterisations for crystalline Th-BTBT-C_{10} and BTBT-C_{10} transistors. a,**b**, Temperature-dependent transfer curves of the Th-BTBT-C_{10} and BTBT-C_{10} transistors. **c**,**d**, Extracted σ_{4p} and Hofstein mobility $\mu_{4p}^{Hofs.}$ for Ph-BTBT-C_{10}, Th-BTBT-C_{10}, and BTBT-C_{10} transistors.

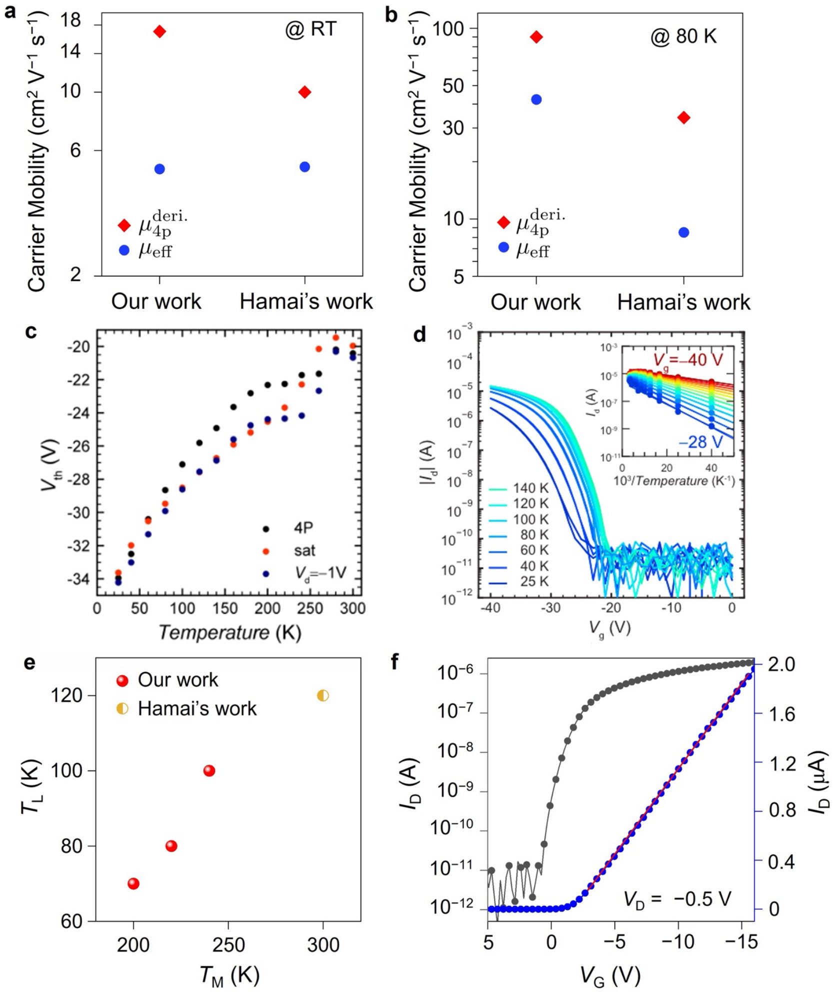

Supplementary Fig. 21 | Comparison of the electrical characteristics of Ph-BTBT-C_{10} transistors in ref. [15] and our work. a,**b**, $\mu_{4p}^{deri.}$ and μ_{eff} in Hamai's work and in our work at room temperature (RT) and 80 K, respectively. The carrier mobility was extracted using the same derivative method. **c**, Threshold voltage (V_T)–temperature (T) dependence in Hamai's work. Note that the thickness of SiO_2 in Hamai's and our work are 100 nm and 50 nm, respectively. **d**, Temperature-dependent transfer curves plotted as a function of V_G in Hamai's work. **e**, Lowest temperature (T_L) where the metallic transport can be maintained in our work and Hamai's work. T_M represents the temperature where the Ph-BTBT-C_{10} transistors were measured initially. **f**, Transfer curves of a Ph-BTBT-C_{10} transistor with transferred electrodes. The dielectric is 100 nm-thick SiO_2. Panels **c** and **d** were reproduced from ref.[15] with permission from Physical Review Materials.

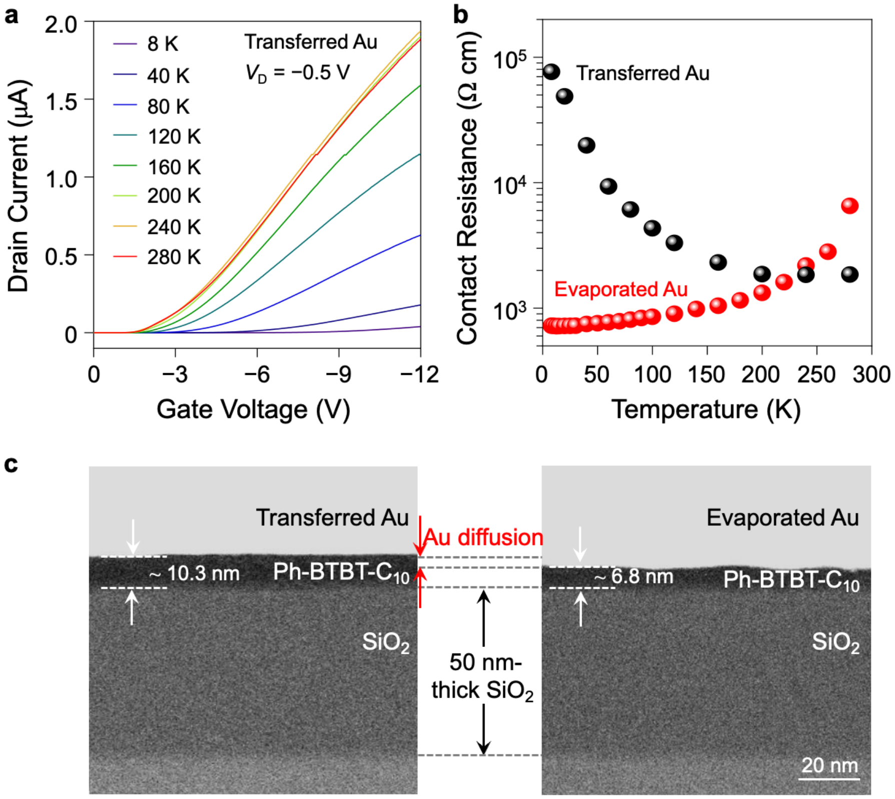

Supplementary Fig. 22 | Electrical characterisations for a Ph-BTBT-C_{10} transistor with transferred electrodes and corresponding electrode/semiconductor interface properties. a, Temperature-dependent transfer curves of a transferred-Au device. The channel length and width were 220 and 100 μm, respectively. **b**, Comparison of contact resistance for evaporated and transferred devices. **c**, STEM images at the electrode/semiconductor interface for transferred and evaporated samples.

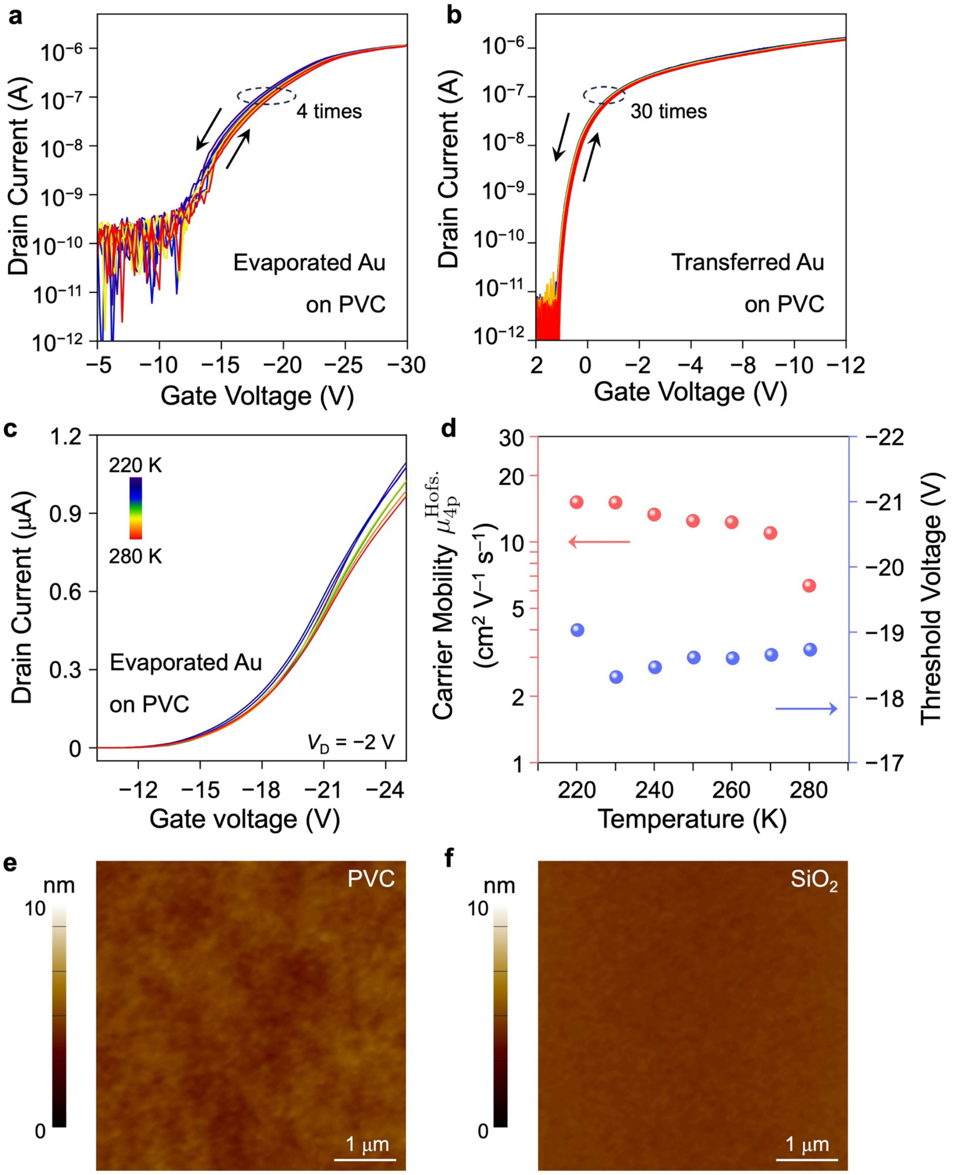

Supplementary Fig. 23 | Device performance of a Ph-BTBT-C_{10} transistor with PVC-modified semiconductor/dielectric interface. a,**b**, Consecutive scanning transfer curves of Ph-BTBT-C_{10} OFETs employing evaporated and transferred Au electrodes on PVC-modified dielectric. **c**, Temperature-dependent transfer curves of a Ph-BTBT-C_{10} device using PVC as dielectric. **d**, The value of $\mu_{4p}^{Hofs.}$ and V_T plotted as a function of temperatures. **e**,**f**, AFM images of the PVC and SiO_2.

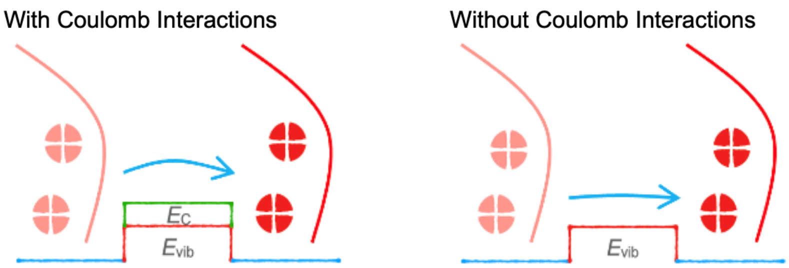

Supplementary Fig. 24 | Hole-hole scattering-induced barrier height increase. The barrier height is increased by the hole-hole Coulomb interaction.

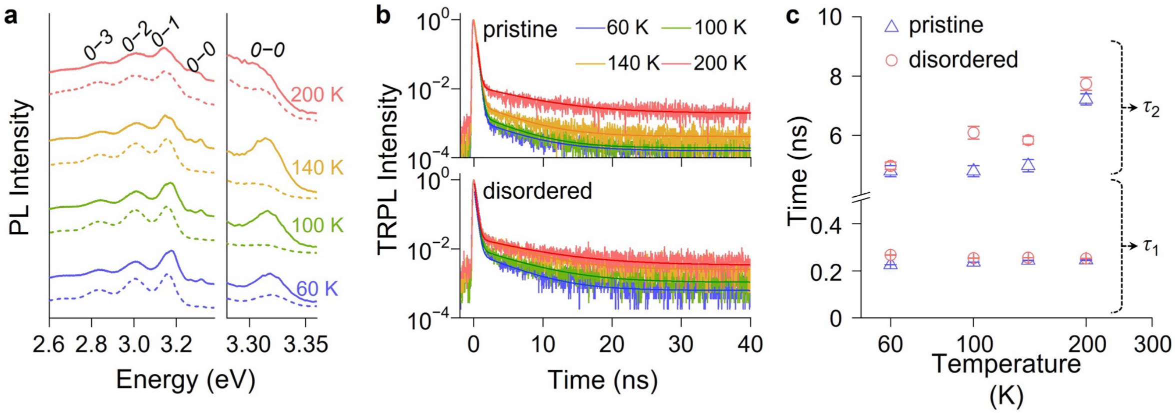

Supplementary Fig. 25 | Photoluminescence spectroscopy (PL) and time-resolved PL (TRPL) characterisations for Ph-BTBT-C_{10} films. a, Temperature-dependent PL spectra for pristine Ph-BTBT-C_{10} (dashed lines) and disordered Ph-BTBT-C_{10} (solid lines). **b**, Temperature-dependent TRPL spectra. **c**, The extracted decay times, representing the density of disorder existing in the Ph-BTBT-C_{10} films. The TRPL decay curve is fitted with thermalized exponential function as $I = A_1\exp(-t/\tau_1) + A_2\exp(-t/\tau_2) + C$, where A_1, A_2 and τ_1, τ_2 are the corresponding weight fraction and decay times, respectively, C is offset parameter relative to PL intensity. The error bars originate from the exponential fitting uncertainty and represent one standard deviation.

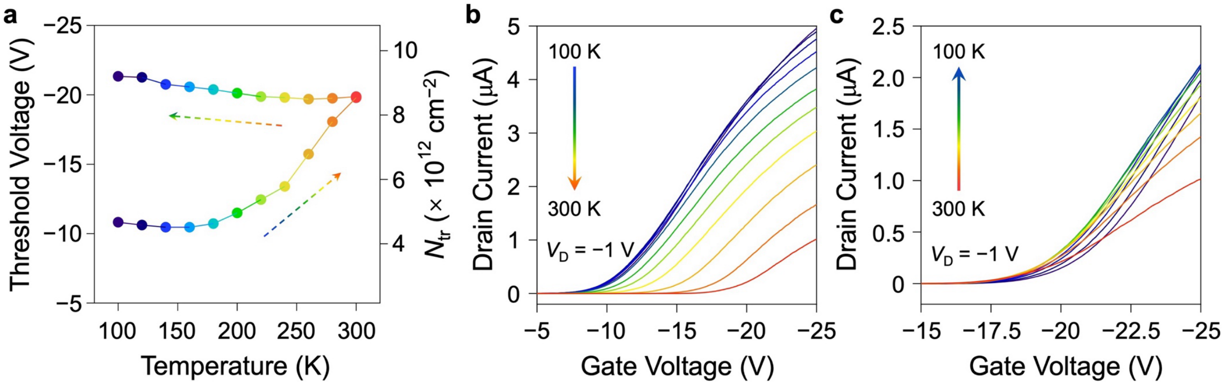

Supplementary Fig. 26 | Threshold voltage shift and corresponding transfer curves of a Ph-BTBT-C_{10} device. a, The threshold voltage plotted as a function of temperature under a cycle test from 100 K to 300 K and back to 100 K. **b**,**c**, Corresponding temperature-dependent transfer curves from 100 K to 300 K and from 100 K to 300 K, respectively.

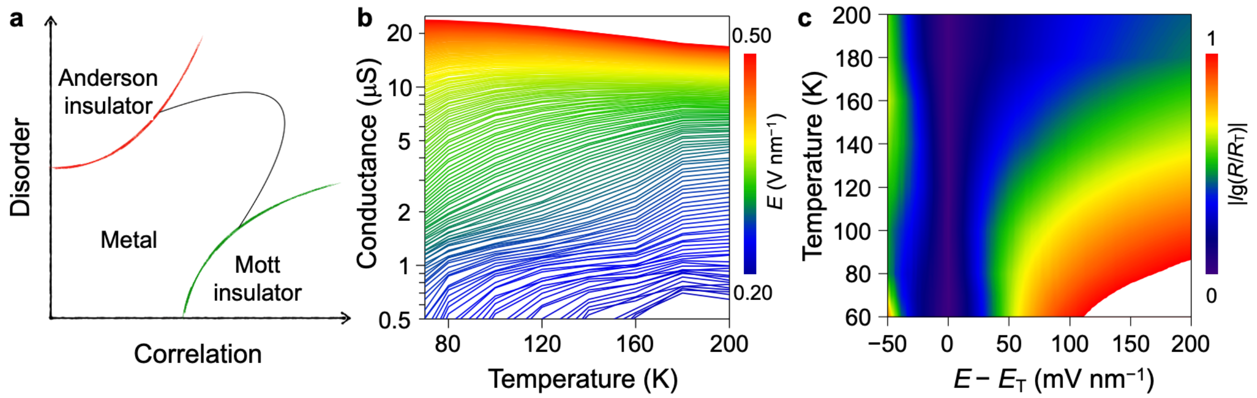

Supplementary Fig. 27 | Phase diagram of Anderson-Hubbard model and temperature-dependent conductance of the Ph-BTBT-C_{10} with introduced disorder. a, Phase diagram of Anderson-Hubbard model, showing the boundaries between metal and Anderson insulator, and between metal and Mott insulator. **b**, Temperature dependence of the conductance under varying electric fields (E). **c**, Electric field-temperature phase diagram for $|\lg(R/R_T)|$, revealing the "fan-shape" structure that is widely observed for quantum criticality for the metal-insulator transition (MIT).

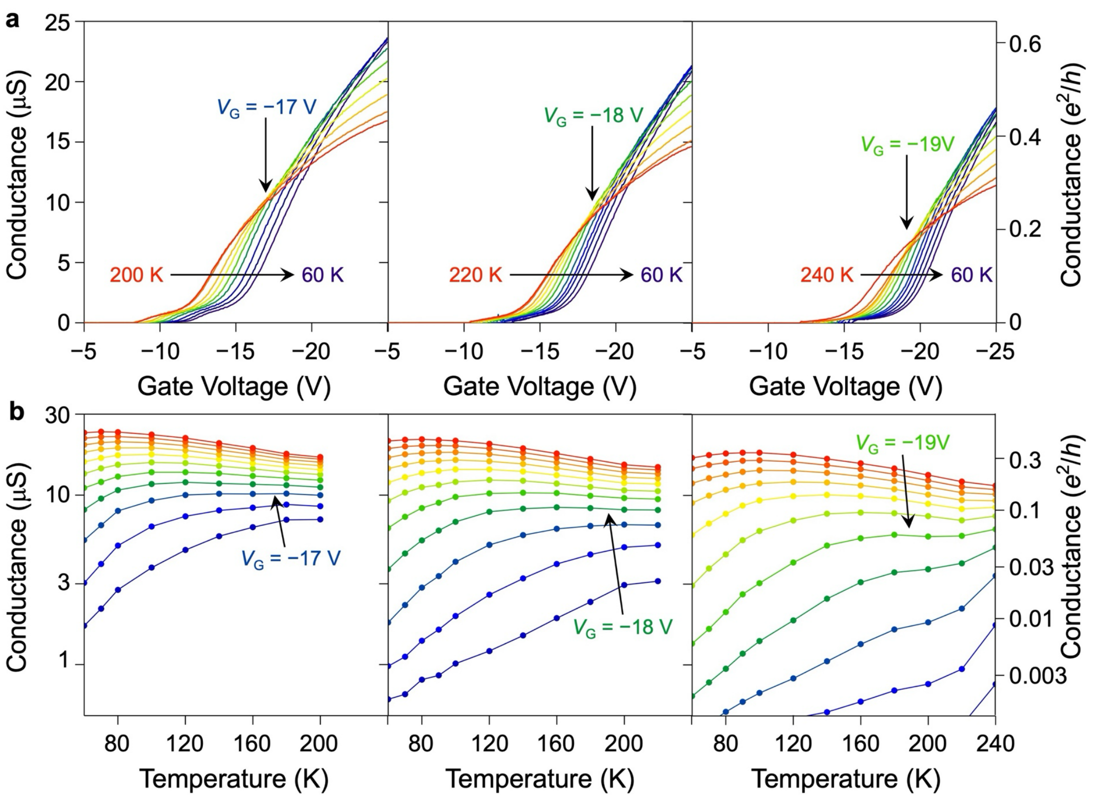

Supplementary Fig. 28 | Disorder-induced MIT in HTH Ph-BTBT-C_{10}. a, Temperature-dependent transfer curves of the same Ph-BTBT-C_{10} transistor with different disorder densities, measured from high to low temperatures. The disorder was introduced by electrical operations at different temperatures (200 K, 220 K, and 240 K, respectively). **b**, Temperature dependence of sheet conductance of the Ph-BTBT-C_{10} sample with various disorder densities.

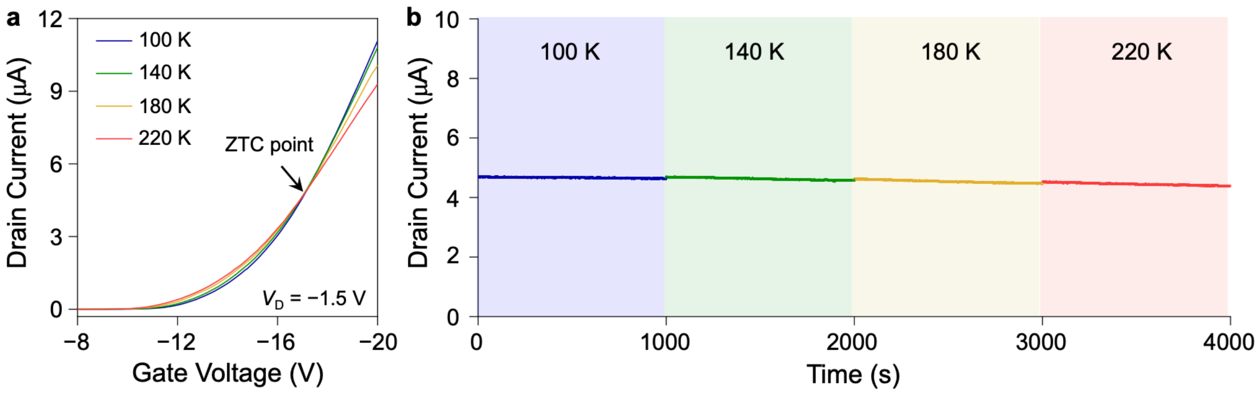

Supplementary Fig. 29 | A temperature-immune OFET. a, Temperature-dependent transfer curves of a Ph-BTBT-C_{10} device, displaying a typical zero-temperature coefficient point at V_G = −17 V. The device was measured from high to low temperature (from 220 K to 100 K). **b**, Bias stress effect tests under different temperatures, where V_G = −17 V and V_D = −1.5 V. Purple, green, yellow, and pink represent 100 K, 140 K, 180 K, and 220 K, respectively.

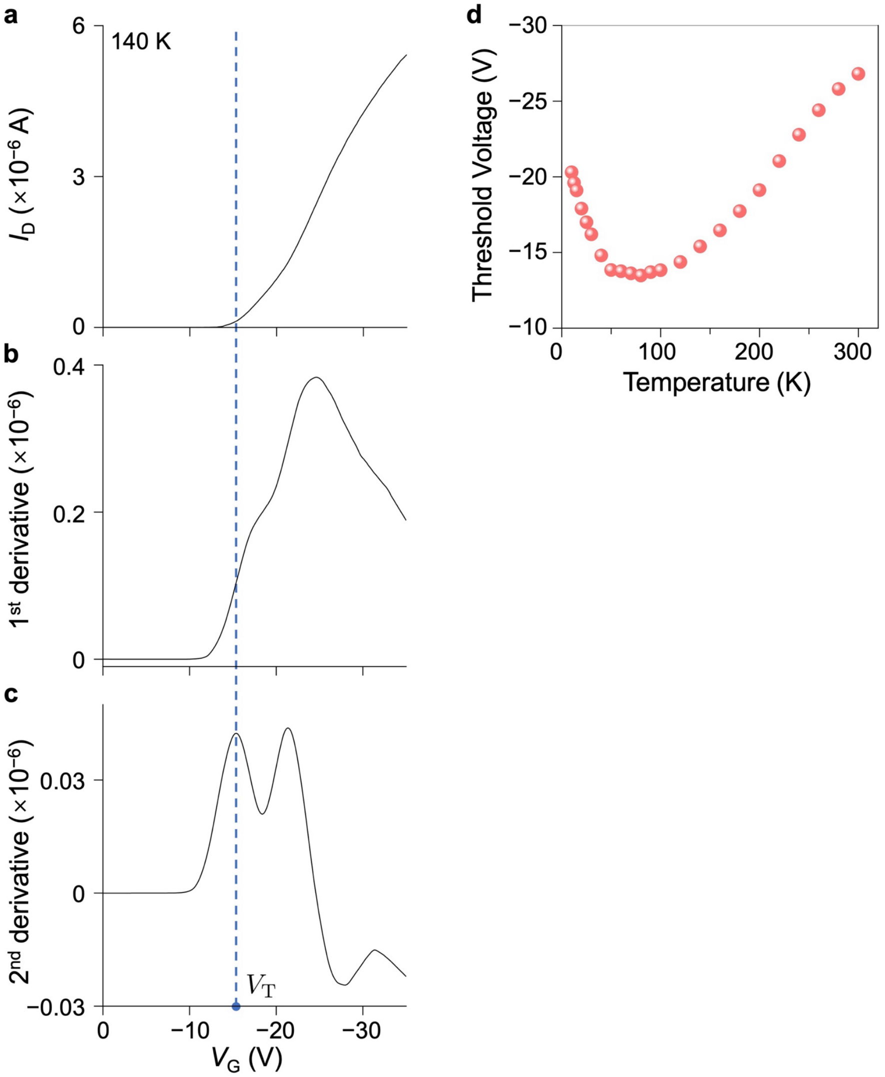

Supplementary Fig. 30 | Threshold voltage (V_T) extraction in OFETs. a, Transfer curve at 140 K. **b**, The first derivative ($g_m = \frac{dI_D}{dV_G}$) of the transfer curve. **c**, The second derivative ($\frac{dg_m}{dV_G}$) of the transfer curve. **d**, The threshold voltages extracted using the second derivative method.

Supplementary Table 1 | Calculated electronic structural parameters and transport parameters of Ph-BTBT-C_{10}, Th-BTBT-C_{10}, and BTBT-C_{10}.

Materials			Ph-BTBT-C_{10}	Th-BTBT-C_{10}	BTBT-C_{10}
Maximum DCD ($\times 10^{-3}$ e Å^{-1})			3.51	2.36	0.17
Binding energy (eV)		interlayer	0.36	0.29	0.18
		intralayer	2.35	2.29	1.89
vdW gap (Å)			2.28	2.90	3.50
Transfer integrals (meV)	Out-of-plane	J_c	7	6	2
	In-plane	J_1	43	35	61
		J_2	137	128	146
		J_3	131	123	139

Supplementary Table 2 | Summary of undoped high-mobility organic semiconductors and MoS_2 in literatures.

Semiconductor	Crystallinity	Thickness	Device Geometry	Gate Dielectric	Mobility Extraction Method	V_D (V)	μ_{FET}[a] ($cm^2 V^{-1} s^{-1}$)	T_1[b] (K)	G_{sheet}[c] (μS)	σ[d] ($S cm^{-1}$)	T_2[e] (K)	V_G[f] (V)	Related Figs.	Refs.
Ph-BTBT-C_{10}	single crystal	10.4 nm	BGTC[g]	SiO_2	4p (Hofs.)	−1	141	8	122	245	8	−35	2d,3c,S14	our work
C_8-BTBT	single crystal	3 nm	BGTC	BN	4p (deri.)	–	25	160	16	53	200	−75	2d,3c,S14	ref.[1]
C_8-BTBT	single crystal	–	BGTC	PMMA/SiO_2	2p	–	8.6	100	–	–	100	−40	–	ref.[23]
Pentacene	single crystal	3 nm	BGTC	BN	2p	−2	5.2	110	1.5	5	110	−50	2d,S14	ref.[20]
Rubrene	single crystal	2 μm	BGBC[h]	vacuum	2p	−6	34	190	38[i,j]	–	–	–	–	ref.[47]
Rubrene-d_{28}	single crystal	–	BGBC	vacuum	4p (deri.)	−1	45	90	0.26	–	90	−60	3c	ref.[25]
Rubrene	single crystal	–	BGBC	vacuum	4p (deri.)	−5	30	180	0.14	–	240	−40	S14	ref.[16]
TIPS-Pentacene	polycrystal	–	TGBC[k]	Cytop	2p	−30	6	10	0.43	4.3	140	−30	2d,S14	ref.[17]
DNTT	single crystal	< 1 μm	BGBC	vacuum	2p	–	10	270	0.09	–	280	−40	S14	ref.[24]
DPA	single crystal	20 nm	BGTC	OTS/SiO_2	2p		11	200	–	–	200	−80	–	ref.[21]
CI_2-NDI	single crystal	5.6 μm	BGBC	vacuum	4p (deri.)	10	11.2	115	0.09	–	150	50	S14	ref.[26]
TIPS-TAP	polycrystal	–	BGTC	AlO_x/SiO_2	2p	50	12.5	210	–	–	250	50	–	ref.[19]
F_2-TCNQ	single crystal	–	BGBC	vacuum	4p (deri.)	–	25	154	–	–	–	–	3c	ref.[18]
2,9-DPh-DNTT	single crystal	200 nm	BGTC	Cytop/SiO_2	2p	−30	18	246	1.8	–	246	−30	S14	ref.[22]
TMTSF	single crystal	bulk	BGBC	vacuum	4p (deri.)	–	6	160	–	–	–	–	3c	ref.[48]
PhC_2−BQQDI	single crystal	–	BGTC	AL-X601 /SiO_2	4p	–	6.7	150	3.6	–	190	65	3c,S14	ref.[49]
PDIF-CN_2	single crystal	–	BGBC	vacuum	4p (deri.)	–	4.1	215	–	–	–	–	3c	ref. [50]
MoS_2	rippled crystal	bilayer	BGTC	SiN_x	4p (deri.)	–	900[i]	–	–	1017[i]	–	–	2d	ref. [51]
MoS_2	single crystal	monolayer	BGTC	BN/SiO_2	4p (Hall)	–	1038	5	–	–	–	–	3c	ref.[52]

[a]Maximum field-effect mobility. [b]Temperature, at which the maximum field-effect mobility was achieved. [c]Maximum sheet conductance. [d]Maximum conductivity measured at various temperatures. [e]Temperature, at which the maximum sheet conductance/conductivity was achieved. [f]Gate voltage at which the maximum conductance/conductivity was achieved. [g]Bottom-gate top-contact. [h]Bottom-gate bottom-contact. [i]Values exacted from devices measured only at 300 K. [j]Value measured using admittance spectroscopy method. [k]Top-gate bottom-contact.

Supplementary Table 3 | Summary of threshold voltage in high-mobility OFETs.

Organic Semiconductor	Gate Dielectric	Electrode Fabrication	Extraction Method	μ_{FET}[a] ($cm^2 V^{-1} s^{-1}$)	V_G^{max} (V)	V_T (T)[b] (V(K))	V_T shift (V)	Refs.
Ph-BTBT-C_{10}	SiO_2	Evap. Au	4p (Hofs.)	141	−35	−21.3 (8)	10	Our work
Ph-BTBT-C_{10}	SiO_2	Evap. Au	4p (deri.)	34	−40	−34 (20)	14	ref.[1]
Ph-BTBT-C_{10}	SiO_2	Trans. Au	2p	15.5*	−16	−1.7 (RT)	–	Our work
Ph-BTBT-C_{10}	OTS/SiO_2	Trans. Au	2p	19.9*	−50	−4 (RT)	–	ref.[31]
TIPS-pentacene	Cytop	Evap. Au[c]	2p	6	−30	−19 (20)	14	ref.[17]
C_8-BTBT	PMMA/SiO_2	Carbon	2p	8.6	−60	−19 (20)	11	ref.[23]
Rubrene	vacuum	Evap. Au[c]	4p (deri.)	30	−40	−19 (120)	13	ref.[16]
Pentacene	BN	Trans. Au	2p	5.2	−50	−32 (50)	19	ref.[20]
DPA	OTS/SiO_2	Evap. Au	2p	11	−80	−50 (100)	21	ref.[21]
TIPS-TAP	AlO_x/SiO_2	Evap. Au	2p	12.5	50	25 (100)	13	ref.[19]

[a]Maximum field-effect mobility measured at low temperature. [b]The value of V_T was obtained at the lowest temperature measured in our work and refs.. [c]This device structure is bottom-gate bottom-contact, and others are bottom-gate top-contact. *The sample is only tested at room temperature.

References

1. He, D. *et al.* Ultrahigh mobility and efficient charge injection in monolayer organic thin-film transistors on boron nitride. *Sci. Adv.* **3**, e1701186 (2017).

2. Liu, J. *et al.* High mobility emissive organic semiconductor. *Nat. Commun.* **6**, 10032 (2015).

3. Illig, S. *et al.* Reducing dynamic disorder in small-molecule organic semiconductors by suppressing large-amplitude thermal motions. *Nat. Commun.* **7**, 10736 (2016).

4. Schweicher, G. *et al.* Chasing the “killer” phonon mode for the rational design of low-disorder, high-mobility molecular semiconductors. *Adv. Mater.* **31**, 1902407 (2019).

5. Ferrari, E. *et al.* Interlayer sliding phonon drives phase transition in the Ph-BTBT-10 organic semiconductor. *Chem. Mater.* **35**, 5777–5783 (2023).

6. Iino, H., Usui, T. & Hanna, J. Liquid crystals for organic thin-film transistors. *Nat. Commun.* **6**, 6828 (2015).

7. Xiao, M. *et al.* Anisotropy of charge transport in a uniaxially aligned fused electron-deficient polymer processed by solution shear coating. *Adv. Mater.* **32**, 2000063 (2020).

8. Sun, H. *et al.* Unidirectional coating technology for organic field-effect transistors: materials and methods. *Semicond. Sci. Technol.* **30**, 054001 (2015).

9. Diao, Y. *et al.* Solution coating of large-area organic semiconductor thin films with aligned single-crystalline domains. *Nat. Mater.* **12**, 665–671 (2013).

10. Giri, G. *et al.* Tuning charge transport in solution-sheared organic semiconductors using lattice strain. *Nature* **480**, 504–508 (2011).

11. Zhang, Z., Peng, B., Ji, X., Pei, K. & Chan, P. K. L. Marangoni-effect-assisted bar-coating method for high-quality organic crystals with compressive and tensile strains. *Adv. Funct. Mater.* **27**, 1703443 (2017).

12. Chen, Y., Zhao, Y., Gao, H. & Zheng, J. Liquid bridge force between two unequal-sized spheres or a sphere and a plane. *Particuology* **9**, 374–380 (2011).

13. Ko, Y. G. & Shin, D. H. Effects of liquid bridge between colloidal spheres and evaporation temperature on fabrication of colloidal multilayers. *J. Phys. Chem. B* **111**, 1545–1551 (2007).

14. Choi, H. H., Cho, K., Frisbie, C. D., Sirringhaus, H. & Podzorov, V. Critical assessment of charge mobility extraction in FETs. *Nat. Mater.* **17**, 2–7 (2018).

15. Hamai, T., Inoue, S., Arai, S. & Hasegawa, T. Trap-state suppression and band-like transport in bilayer-type organic semiconductor ultrathin single crystals. *Phys. Rev. Mater.* **4**, 074601 (2020).

16. Podzorov, V. *et al.* Intrinsic charge transport on the surface of organic semiconductors. *Phys. Rev. Lett.* **93**, 086602 (2004).

17. Sakanoue, T. & Sirringhaus, H. Band-like temperature dependence of mobility in a solution-processed organic semiconductor. *Nat. Mater.* **9**, 736–740 (2010).

18. Krupskaya, Y., Gibertini, M., Marzari, N. & Morpurgo, A. F. Band-like electron transport with record-high mobility in the TCNQ family. *Adv. Mater.* **27**, 2453–2458 (2015).

19. Xu, X. *et al.* Electron mobility exceeding 10 $cm^2 V^{-1} s^{-1}$ and band-like charge transport in solution-processed n-channel organic thin-film transistors. *Adv. Mater.* **28**, 5276–5283 (2016).

20. Zhang, Y. *et al.* Probing carrier transport and structure-property relationship of highly ordered organic semiconductors at the two-dimensional limit. *Phys. Rev. Lett.* **116**, 016602 (2016).

21. Ji, D. *et al.* Band-like transport in small-molecule thin films toward high mobility and ultrahigh detectivity phototransistor arrays. *Nat. Commun.* **10**, 12 (2019).

22. Takimiya, K., Bulgarevich, K., Horiuchi, S., Sato, A. & Kawabata, K. Bandlike versus temperature-independent carrier transport in isomeric diphenyldinaphtho[2,3- *b* :2′,3′- *f*]thieno[3,2- *b*]thiophenes. *ACS Mater. Lett.* **4**, 675–681 (2022).

23. Liu, C. *et al.* Solution-processable organic single crystals with bandlike transport in field-effect transistors. *Adv. Mater.* **23**, 523–526 (2011).

24. Xie, W. *et al.* Temperature-independent transport in high-mobility dinaphtho-thieno-thiophene (DNTT) single crystal transistors. *Adv. Mater.* **25**, 3478–3484 (2013).

25. Xie, W. *et al.* High-mobility transistors based on single crystals of isotopically substituted rubrene-d_{28}. *J. Phys. Chem. C* **117**, 11522–11529 (2013).

26. He, T. *et al.* Crystal step edges can trap electrons on the surfaces of n-type organic semiconductors. *Nat. Commun.* **9**, 2141 (2018).

27. Hutsch, S., Panhans, M. & Ortmann, F. Charge carrier mobilities of organic semiconductors: ab initio simulations with mode-specific treatment of molecular vibrations. *Npj Comput. Mater.* **8**, 228 (2022).

28. Tsurumi, J. *et al.* Coexistence of ultra-long spin relaxation time and coherent charge transport in organic single-crystal semiconductors. *Nat. Phys.* **13**, 994–998 (2017).

29. Xu, Y. *et al.* Essential effects on the mobility extraction reliability for organic transistors. *Adv. Funct. Mater.* **28**, 1803907 (2018).

30. Choi, H. H. *et al.* Accurate extraction of charge carrier mobility in 4-probe field-effect transistors. *Adv. Funct. Mater.* **28**, 1707105 (2018).

31. Yao, J. *et al.* Impact of surface free energy on two-dimensional crystallization. *Sci. CHINA Mater.* **66**, (2023).

32. Sun, L. *et al.* Modulating contact properties by molecular layers in organic thin-film transistors. *Electron* **1**, e7 (2023).

33. Jiang, C. *et al.* Printed subthreshold organic transistors operating at high gain and ultralow power. *Science* **363**, 719–723 (2019).

34. Hestand, N. J. & Spano, F. C. Expanded theory of H- and J-molecular aggregates: the effects of vibronic coupling and intermolecular charge transfer. *Chem. Rev.* **118**, 7069–7163 (2018).

35. Siegrist, T. *et al.* Disorder-induced localization in crystalline phase-change materials. *Nat. Mater.* **10**, 202–208 (2011).

36. Finlayson, D. M. Metal-insulator transition in InP. *Philos. Mag. Lett.* **61**, 293–296 (1990).

37. Mott, N. F. Metal-insulator transitions. *Contemp. Phys.* **14**, 401–413 (1973).

38. Smoleński, T. *et al.* Signatures of Wigner crystal of electrons in a monolayer semiconductor. *Nature* **595**, 53–57 (2021).

39. Zhou, Y. *et al.* Bilayer Wigner crystals in a transition metal dichalcogenide heterostructure. *Nature* **595**, 48–52 (2021).

40. Li, T. *et al.* Continuous Mott transition in semiconductor moiré superlattices. *Nature* **597**, 350–354 (2021).

41. Ahn, S. & Das Sarma, S. Disorder-induced two-dimensional metal-insulator transition in moiré transition metal dichalcogenide multilayers. *Phys. Rev. B* **105**, 115114 (2022).

42. Kravchenko, S. V. & Sarachik, M. P. Metal-insulator transition in two-dimensional electron systems. *Rep. Prog. Phys.* **67**, 1–44 (2004).

43. Bogdanovich, S. & Popović, D. Onset of glassy dynamics in a two-dimensional electron system in silicon. *Phys. Rev. Lett.* **88**, 236401 (2002).

44. Di Sante, D., Fratini, S., Dobrosavljević, V. & Ciuchi, S. Disorder-driven metal-insulator transitions in deformable lattices. *Phys. Rev. Lett.* **118**, 036602 (2017).

45. Boudinet, D. *et al.* Contact resistance and threshold voltage extraction in n-channel organic thin film transistors on plastic substrates. *J. Appl. Phys.* **105**, 084510 (2009).

46. Wong, H.-S., White, M. H., Krutsick, T. J. & Booth, R. V. Modeling of transconductance degradation and extraction of threshold voltage in thin oxide MOSFET's. *Solid-State Electron.* **30**, 953–968 (1987).

47. Blülle, B., Troisi, A., Häusermann, R. & Batlogg, B. Charge transport perpendicular to the high mobility plane in organic crystals: Bandlike temperature dependence maintained despite hundredfold anisotropy. *Phys. Rev. B* **93**, 035205 (2016).

48. Xie, H., Alves, H. & Morpurgo, A. F. Quantitative analysis of density-dependent transport in tetramethyltetraselenafulvalene single-crystal transistors: Intrinsic properties and trapping. *Phys. Rev. B* **80**, 245305 (2009).

49. Kumagai, S. *et al.* Coherent electron transport in air-stable, printed single-crystal organic semiconductor and application to megahertz transistors. *Adv. Mater.* **32**, 2003245 (2020).

50. Minder, N. A., Ono, S., Chen, Z., Facchetti, A. & Morpurgo, A. F. Band-like electron transport in organic transistors and implication of the molecular structure for performance optimization. *Adv. Mater.* **24**, 503–508 (2012).

51. Ng, H. K. *et al.* Improving carrier mobility in two-dimensional semiconductors with rippled materials. *Nat. Electron.* **5**, 489–496 (2022).

52. Cui, X. *et al.* Multi-terminal transport measurements of MoS_2 using a van der Waals heterostructure device platform. *Nat. Nanotechnol.* **10**, 534–540 (2015).
